# The mitochondrial citrate transporter, CIC, is essential for mitochondrial homeostasis

**DOI:** 10.18632/oncotarget.714

**Published:** 2012-10-20

**Authors:** Olga Catalina-Rodriguez, Vamsi K. Kolukula, York Tomita, Anju Preet, Ferdinando Palmieri, Anton Wellstein, Stephen Byers, Amato J. Giaccia, Eric Glasgow, Chris Albanese, Maria Laura Avantaggiati

**Affiliations:** ^1^ Department of Oncology, Lombardi Comprehensive Cancer Center, Georgetown University, Washington, DC, USA; ^2^ Department of Pharmaco-Biology, University of Bari, Bari, Italy; ^3^ Department of Radiation Oncology, Stanford University School of Medicine, Stanford, CA, USA

**Keywords:** SLC25A1, CIC, citrate, cancer, mitochondria, autophagy, Di-George Syndrome

## Abstract

Dysregulation of the pathways that preserve mitochondrial integrity hallmarks many human diseases including diabetes, neurodegeration, aging and cancer. The mitochondrial citrate transporter gene, SLC25A1 or CIC, maps on chromosome 22q11.21, a region amplified in some tumors and deleted in developmental disorders known as velo-cardio-facial- and DiGeorge syndromes. We report here that in tumor cells CIC maintains mitochondrial integrity and bioenergetics, protects from mitochondrial damage and circumvents mitochondrial depletion via autophagy, hence promoting proliferation. CIC levels are increased in human cancers and its inhibition has anti-tumor activity, albeit with no toxicity on adult normal tissues. The knock-down of the CIC gene in *zebrafish* leads to mitochondria depletion and to proliferation defects that recapitulate features of human velo-cardio-facial syndrome, a phenotype rescued by blocking autophagy. Our findings reveal that CIC maintains mitochondrial homeostasis in metabolically active, high proliferating tissues and imply that this protein is a therapeutic target in cancer and likely, in other human diseases.

## INTRODUCTION

The solute family member SLC25A1, or CIC, belongs to a family of ion transporters embedded in the inner mitochondrial membrane, and whose defects have been indirectly linked to various human diseases [1,2,3]. The human CIC gene maps on chromosome 22.q11.2. Several chromosomal translocations and amplifications involving 22q11.2 have been described in various tumors, while microdeletions of this region give raise to developmental disorders known as velo-cardio-facial- (VCFS), DiGeorge syndromes (DGS) as well as to some forms of schizophrenia [4,5]. CIC expression is induced by insulin and by inflammation, its activity is lost in type-1 diabetes, and early studies also suggested increased activity in tumors of the liver [6,7,8,9]. Furthermore, mutations of a member of the tricarboxylate transporter family in fruit fly, *INDY*, promote longevity [10], thus suggesting that the citrate transport pathway also controls life-span. Although CIC has been studied in *yeast*, there is currently little information on the effects of the human protein on proliferation.

**Figure 1:**
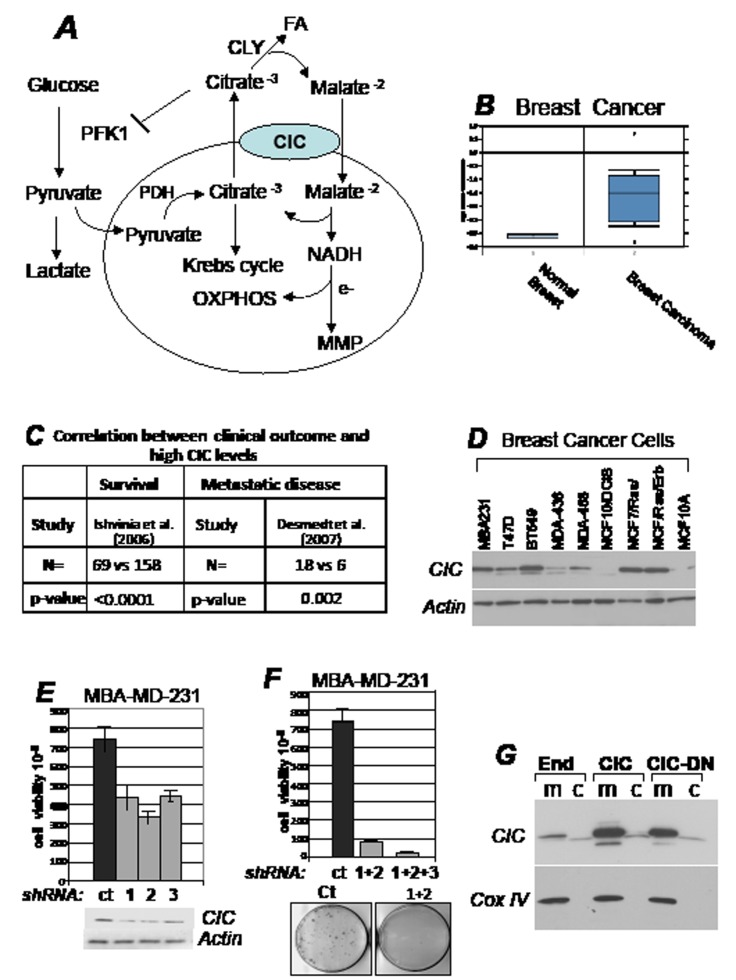
CIC is required for tumor proliferation A. Role of CIC in metabolism (see also text for explanation). CIC catalyzes the electroneutral exchange across the inner mitochondrial membrane of the tricarboxylate citrate plus a proton for the dicarboxylate malate. Once in the cytoplasm citrate is cleaved by citrate lyase (CLY), providing a source for fatty acids (FA) synthesis and producing malate that is imported in the mitochondria. Here, malate oxidation generates NADH, which donates its electrons to the transport chain thereby maintaining oxidative phosphorylation (OXHOPHOS) and mitochondrial membrane potential (MMP). Cytoplasmic citrate can also act as an allosteric inhibitor of Phosphofructokinase-1 (PK1), a key glycolytic enzyme. Glycolysis in the cytoplasm generates a second key substrate, pyruvate that is either converted into lactate to produce ATP in an anaerobic reaction, or it is transported into the mitochondria, converted by Pyruvate Dehydrogenase (PDH) into Acetyl-Coenzyme A, recondensed to citrate, which then supports the Krebs cycle. These activities linked to citrate, should place CIC at a nodal point in regulation of mitochondrial activity and metabolism. B. CIC mRNA expression levels in human normal or breast tumor tissues. Data were obtained from the Oncomine database [49], by selecting a minimal threshold p-value of 0.0005. C. Negative correlation between CIC expression levels and the development of metastatic disease in breast cancer. *N* indicates the total number of patients. In one study conducted [50], 69 patients with elevated CIC levels were dead after 5 years, *versus* 158 patients with lower levels, who were still alive. In a second study [51], 18 patients with elevated CIC levels developed metastasis after 5 years, *versus* 6 with lower CIC levels who remained metastasis free. p values are indicated in each panel. D. CIC expression levels in the breast cancer cell lines indicated at the top of each panel. E-F. Clonogenic assays performed in MBA-MD-231 cells. Cells were transfected with control shRNA vector (indicated as ct), or with vectors harboring three CIC shRNA (shRNA 1, 2, 3), each harboring a puromycin resistance gene. Vectors were transfected individually (panel E) or in different combinations (panel F) and proliferation was assessed after one week of antibiotic selection. Representative CIC expression levels relatively to actin levels are shown in panel E (bottom panel), and representative plates derived from the combination transfection experiments are shown at the bottom of panel F. G. Mitochondrial (m) and cytoplasmic (c) fractions derived from 293T cells transfected with the pcDNA4/TO control vector, or expressing CIC or CIC-DN. The expression levels of CIC and COX IV, used as a mitochondrial marker, are shown.

Mechanistically, CIC promotes the efflux across the mitochondria of tricarboxylic citrate in exchange for dicarboxylic cytosolic malate (the citrate malate antiport), leading to important metabolic adjustments (Figure 1A) [1,11]. Citrate, which is produced predominantly in the mitochondria via glucose-derived pyruvate, serves as a key substrate for the generation of energy and as an allosteric modulator of several enzymes as well. In the mitochondria citrate is oxidized *via* the Krebs cycle and Oxidative Phosphorylation (OXOPHOS), while in the cytoplasm it supports lipid synthesis and blunts glycolysis by inhibiting phosphofructokinase-1 (PFK1) [12,13]. Additionally, the entry of malate into the mitochondria in exchange for citrate stimulates OXOPHOS and is coupled to the transport of one proton [13]. This modality of exchange maintains both electroneutrality across the mitochondrial membrane and OXOPHOS, because malate enters into the Krebs cycle in place of citrate. Therefore, the citrate export pathway might impact upon mitochondrial respiration, upon the stability of mitochondrial membrane potential (MMP), -which is directly linked to the proton flux and to the activity of the electron transport chain-, as well as on glycolysis and on lipogenesis.

The majority of tumor cells display alterations in metabolism relatively to normal cells that are intimately connected with cancer development, progression and invasiveness [14]. These metabolic changes center in large part upon the different utilization of citrate compared to normal cells. Indeed, it has been proposed that in cancer cells citrate is predominantly exported out the mitochondria *via* CIC, being subsequently cleaved in the cytoplasm via citrate lyase (CLY) to support lipid, Acetyl-Coenzyme A and macromolecular biosynthesis [15,16,17]. This switch from mitochondrial to cytoplasmic metabolism of citrate is thought to account for the acquisition of the lipogenic phenotype that, together with the high rates of aeorobic glycolysis (the Warburg effect), represents a hallmark of many cancer cells [18,19]. The glycolytic behavior of tumors was initially attributed to impairment of mitochondrial respiratory activity. This hypothesis has been recently revisited in light of evidence showing that the mitochondria of cancer cells retain some extent of respiratory capacity [18]. In addition, recent data demonstrates that the tumor stroma provides a variety of catabolized nutrients that enables the anabolic growth of tumor cells by enhancing their mitochondrial activity [14,20]. Thus, albeit the role of the mitochondria in tumor proliferation has been for a long time undermined by the Warburg hypothesis, it is now clear that these organelles enact essential metabolic circuits needed for tumorigenesis, including OXOPHOS, glutamine and fatty acid oxidation [18].

Nevertheless, mitochondrial DNA mutations, the hypoxic and nutrient restricted microenvironment as well as oncogenic mutations that reduce the activity of mitochondrial enzymes, all contribute to oxidative and respiration stress endured by these organelles [21,22]. Consequently to these alterations, cancer cells produce radical oxygen species (ROS), which increase oxidative burden, but also work to the advantage of tumors because low ROS levels produced by the stroma or within cancer cells promote proliferation [14,22]. This oxidative instability is at the same time also a double edge sword and a vulnerable aspect of cancer cells, given that enhancing ROS production leads to tumor killing and to activation of a specialized form of autophagy, termed mitophagy [23,24]. Although activation of mitophagy in the adjacent stroma feeds cancer cells with nutrients required for proliferation [14], avoidance of mitophagy within the tumor appears instead to be advantageous to cancer growth at least in some conditions [25,26].

Thus, cancer cells must maintain an adequate equilibrium between mitochondrial activity, mitophagy, and ROS production. Results presented here demonstrate that CIC is important for preserving such balance.

## RESULTS

### CIC levels are elevated in human cancers and its activity is required for tumor proliferation *in vitro*

This study stemmed from the finding that the transcription rate of the CIC promoter is enhanced by several oncogenic pathways including mutant forms of p53 and Myc, while it is repressed by the tumor suppressor PTEN (*Palmieri and Avantaggiati, manuscript in preparation*). Consistent with this observation, we then found that CIC mRNA levels are elevated in various cancer cell lines and human tumors correlating with poor survival rates or with the development of metastatic disease (Figure 1BC; Supplementary Figure S1AD). CIC levels were found elevated in many, but not all of the tumor cell lines derived from the NCI50 panel (Figure S1, panels F-I). Significantly, CIC is almost undetectable in a model system of ductal non-invasive carcinoma *in situ* (DCIS) or in pseudo-normal MCF10A mammary cells, compared to invasive MCF10A cells expressing the Ras/Erb B oncogenes, or to MDA-MB-231 and BT549 cell lines (Figure 1D). These observations suggested that CIC expression correlates with aggressiveness. In agreement with this idea, in MBA-MD-231 cells, expression of three different CIC shRNAs, each transfected individually, resulted in a small reduction in CIC protein and in a modest growth inhibition (Figure 1E). However, co-transfection of combination shRNAs completely blunted proliferation (Figure 1F) and clones that survived these experiments again displayed CIC levels nearly similar to control (not shown). Thus, complete CIC inhibition is incompatible with survival of MBA-MD-231 cells.

**Figure 2:**
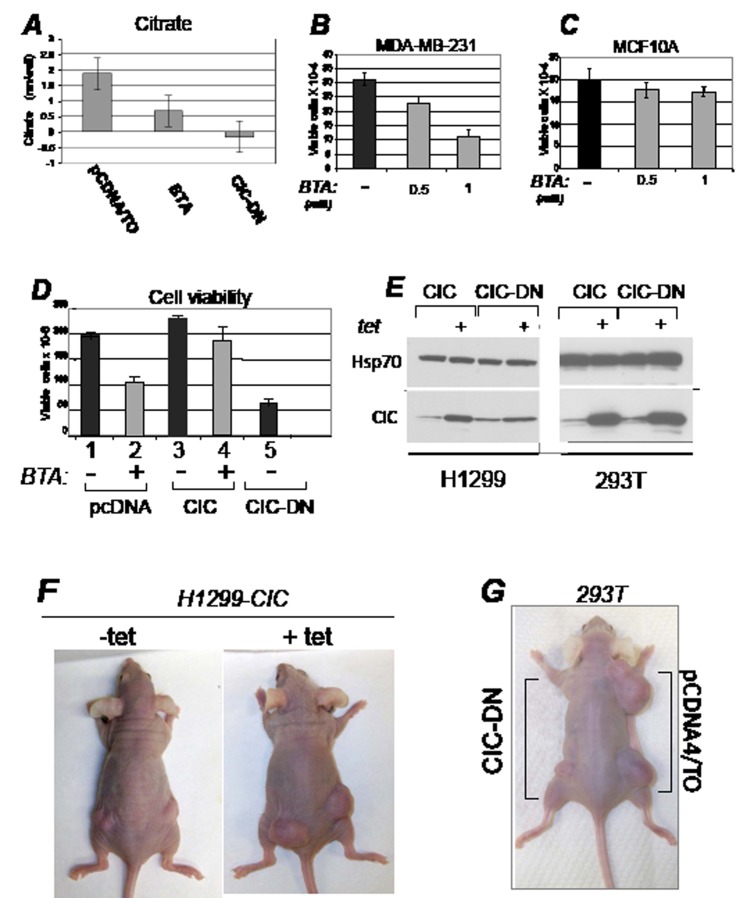
CIC maintains the intracellular pool of citrate and promotes proliferation A. Citrate levels (nM/well) were assessed in 293T cell lines harboring the pcDNA/TO vector and treated with either DMSO or with 1 mM BTA, or in cells expressing CIC-DN. Negative values are due to subtraction of background rates. B-C. Dose dependent growth inhibition of BTA in MDA-MB-231 or MCF10A cells. D. Control H1299 cells (pcDNA/TO), or cells expressing the CIC proteins indicated at the bottom of each panel, were treated with DMSO (-), or with 1 mM BTA (+). Cell viability was assessed with trypan blue exclusion. E. Expression of CIC or CIC-DN in tetracycline inducible stable clones of H1299 or 293T cells employed in this study, in presence or absence of tetracycline. F. Female athymic balbc/ nude mice were injected with H1299 cells expressing inducible CIC in the presence or absence of tetracycline or of vehicle control provided in the water. G. 293T cells stably transfected with the control vector (pCDNA4/TO), or expressing inducible CIC-DN were injected in the right and left flanks of female athymic mice, respectively.

To specifically probe the relevance of the citrate export activity of CIC on proliferation, we used a non-cleavable benzene-tricarboxylate analog (BTA) that suppresses CIC-dependent transport of citrate *in vivo* [6,27]. We also designed a CIC mutant protein where three amino acid residues necessary for citrate export, K190, N194 and R198, were replaced with C190, I194 and C198, respectively [11,28,29]. This mutant (CIC-DN) did not affect mitochondrial localization or embedding in the mitochondrial membrane (Figure 1G; Figure S2AB) but exhibited dominant negative activities, as judged by its ability to lower the concentration of citrate in cells expressing endogenous CIC (Figure 2A). Limited tryptic digestion experiments further showed an impaired ability of CIC-DN to interact with the citrate analog BTA, as it was expected (Figure S2CD). To assess the effects of CIC and of its inhibition on cell growth, several cell lines were treated with BTA or were transfected with the vectors encoding CIC or CIC-DN. In MBA-MD-231, but not in the pseudo-normal MCF10A cells, treatment with BTA decreased proliferation rates in a dose-dependent manner (Figure 2BC). Such differential effects suggested a preferential sensitivity of tumor cells to CIC inhibition, a view supported by studies shown later. Furthermore, over-expression of wild-type CIC modestly but reproducibly enhanced the proliferation potential of H1299 cells, and rescued growth inhibition due to BTA treatment (Figure 2D, compare lane 2 with lane 4). By contrast expression of CIC-DN was detrimental for cell growth (Figure 2D, lane 5).

These results provide the first line of evidence that inhibition of CIC hampers cancer cell proliferation, show that CIC is a target of the anti-proliferative action of BTA *in vivo*, and demonstrate the importance of the citrate export pathway activity of CIC in regulation of growth.

### Inhibition of CIC inhibits tumorigenesis *in vivo*, but is non-toxic to adult normal tissues

**Figure 3:**
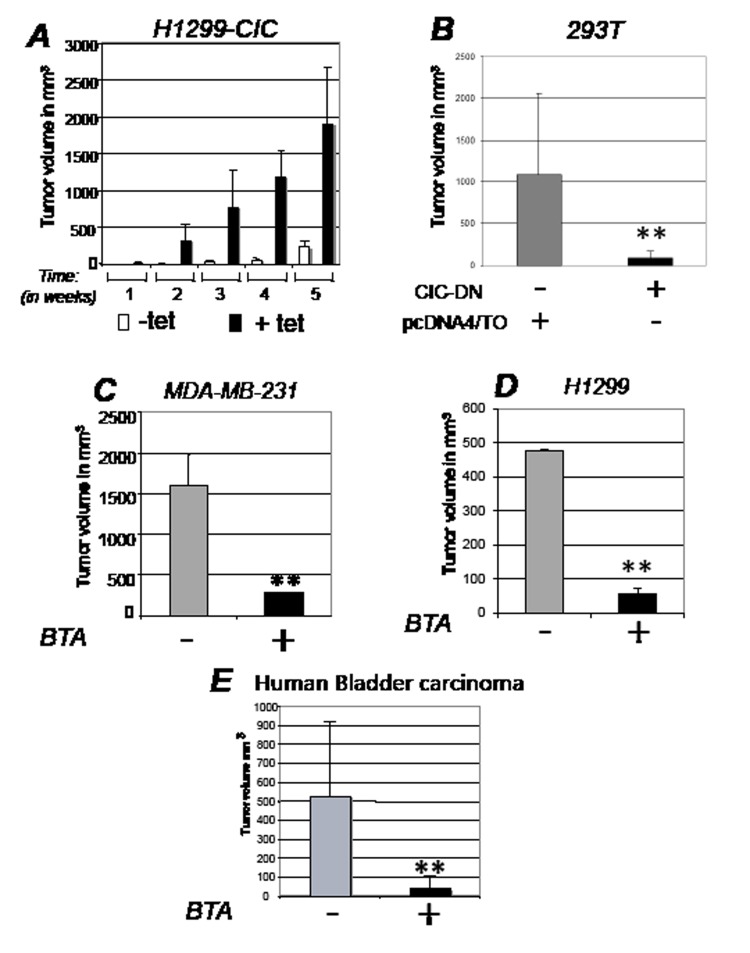
CIC is rate-limiting for tumorigenesis in vivo A. Female athymic balbc/ nude mice were injected with H1299 cells expressing inducible CIC in the presence or absence of tetracycline or of vehicle control as described in Figure 2F and tumor volumes were assessed several weeks after implantation. White bars indicate control group, while black bars show tumor growth in cells expressing CIC. B. Tumor volumes assessed after implantation in nude mice of T293 cells expressing control vector (pCDNA/TO) or dominant negative CIC (CIC-DN). C-E. Tumor volumes in female athymic balbc/ nude mice injected with breast cancer MDA-MB-231 cells (C); with lung cancer H1299 cell lines (D) cells; or with bladder cancer cells derived from T24 cells (E). Tumor volumes derived from mock treated (-) and BTA treated (+) mice are shown. Bars represent standard deviations; asterisk (*) represents p-value <0.05 and double-asterisk (**) represents p-value <0.01.

To explore whether CIC affects tumor development *in vivo*, we next generated stable lung cancer (H1299) and SV40 T-antigen transformed embryonic kidney (293T) cell lines expressing tetracycline inducible CIC or CIC-DN (Figure 2E), and thereby assessed their growth capacity after implantation in immuno-deficient mice. CIC dramatically accelerated the onset and size of tumors, while CIC-DN led to a reduction of growth (Figure 2FG, and Figure 3AB). CIC proteins were detectable in these tumors (Figure S3A), arguing that their opposite effects on proliferation are directly linked to changes in their activity. Similarly to CIC-DN, BTA also exhibited anti-cancer activity in various tumor types, including in aggressive breast cancer cell lines MDA-MB-231, lung cancer cells H1299 as well as in bladder cancers cell lines (Figure 3CE). The anti-tumor effect of BTA observed in MDA-MB-231 cells as a single agent (Figure 3C), is of particular relevance given that these cells are representative of triple receptor negative breast tumors that are often hormone- and chemo- resistant [30]. During the course of these experiments, we also noticed that BTA-treated mice showed no evidence of illness or toxicity. Therefore, we examined potential side effects of this compound in non immuno-compromised animals (Figure S3CE). Mice were randomized and BTA was administered twice a week for five consecutive months. There was a small but statistically significant reduction of the body weight in all of the BTA-treated animals, but animals could be treated for a long period of time without showing any evidence of toxicity. Thus, we conclude that inhibition of CIC activity is non-toxic in the adult normal mouse. By contrast, CIC is rate limiting for tumor progression *in vivo,* and its inhibition has therapeutic potential.

### CIC inhibition leads to mitochondrial dysfunction, destabilizes MMP and enhances glycolysis

We next studied the molecular mechanisms by which CIC affects proliferation. The function of CIC has been primarily linked to glucose-derived fatty acid (FA) synthesis, because of its role in citrate export [11,17]. Therefore we studied the metabolism of D-[1,6-^13^C_2_] glucose *via* NMR mass spectrometry. In support of the proposed role of CIC in promoting *de novo* lipogenesis, both BTA and CIC-DN severely reduced the ability of cells to convert glucose into FA (Figure S4AB). However, the total FA levels, which can be synthesized also from glycerol and amino acids, were modestly reduced at 16 hours after BTA treatment (not sown), but at later time points they were increased (Figure S4CD). Furthermore, incubation of BTA-treated cells with the lipid precurson, palmitic acid was not sufficient to rescue proliferation (not shown). These observations led us to conclude that depletion of lipids unlikely represents the only mechanism by which CIC inhibition affects tumor growth.

**Figure 4:**
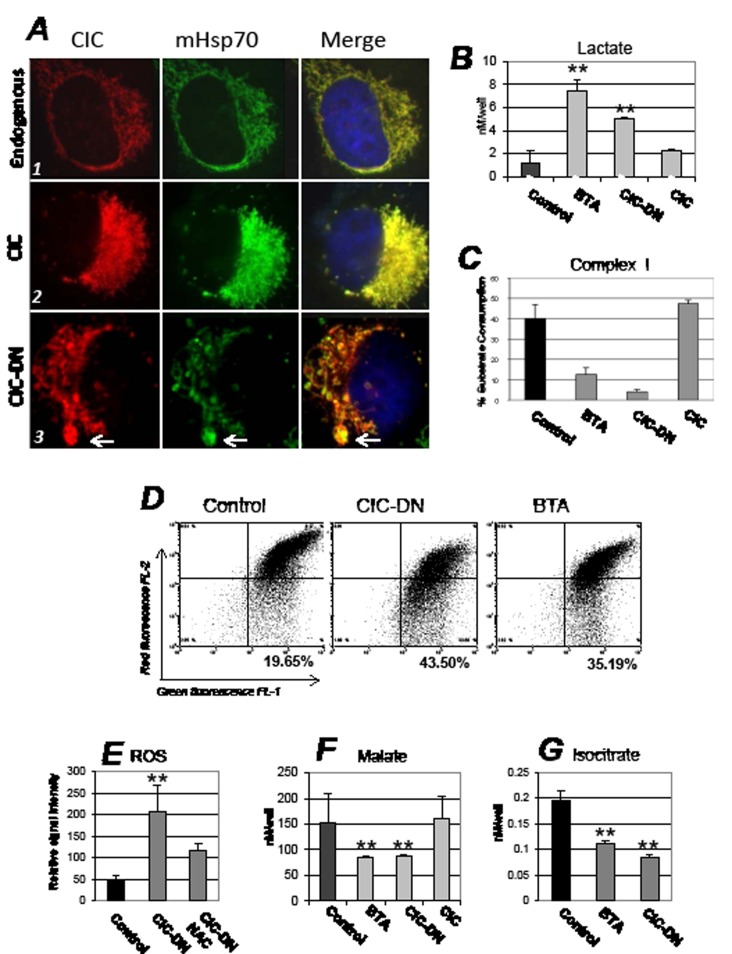
Mitochondrial and metabolic effects arising from CIC inhibition A. H1299 cells (panel set indicated as *1*), or cells expressing CIC (*2*) or CIC-DN (*3*) were stained with anti-CIC (red) or anti-mitochondrial Hsp70 (mHsp70, green) antibodies, and counterstained with DAPI. The DAPI signal is shown only in the merged images. For these experiments cells were harvested 46 hours after tetracycline induction. The arrow in panel A3 indicates mitochondria with enlarged and abnormal morphology (mega-mitochondria). The next set of panels shows measurements of lactate levels (B), of complex I- (C); of mitochondrial membrane potential assessed with a JC1 assay (MMP) (D); of ROS (E); of malate (F); and of isocitrate (G) levels. Measurements were performed in cells treated with DMSO (indicated as control), or with BTA, or expressing CIC or CIC-DN at 16 hours after treatment or tetracycline induction, respectively. In the case of ROS levels, panel D shows a dot plot or red (FL-2) versus green fluorescence (FL-1) of JC1 staining. In mitochondria with intact membrane potential the JC1 dye concentrates into red fluorescent aggregates, while depolarized mitochondria are unable to concentrate the JC1 and exhibit green fluorescence. The percentage of cells with depolarized mitochondrial membrane is shown in bold at the bottom of each panel.

Since CIC is a mitochondrial protein we examined mitochondrial morphology. Immuno-fluorescence experiments showed that in CIC-expressing cells the mitochondria were highly interconnected, while in cells harboring CIC-DN the mitochondrial network was dispersed with fewer and fragmented mitochondria (Figure 4A, indicated by arrow; compare panel *3* with panels *1* and *2*). Mitochondria structure is a direct reflection of metabolic state, and a fragmented phenotype has often been observed in conditions of reduced OXOPHOS and of enhanced glycolysis, as well as a consequence of ROS production [31,32]. This suggested that CIC influences mitochondrial activity and glucose metabolism. Indeed, we found that BTA enhanced the levels of glucose-derived lactate (Figure S4B), and the total levels of lactate were elevated in cells where CIC was inhibited (Figure 4B). In the case of CIC-DN lactate was more prominently increased in the media than inside the cells, perhaps suggesting that this mutant and BTA are not exactly identical in the way they increase glucose flux (Figure 4B; Figure S4E). Consistent with a protective role of CIC on mitochondria activity and OXOPHOS, its inhibition led to a decline of respiratory Complex-I activity (Figure 4C). This was accompanied by a loss of mitochondrial membrane polarity (Figure 4D) and by the production of ROS, which was rescued by the general ROS scavenger, N- acetyl-cysteine (NAC) (Figure 4E).

**Figure 5:**
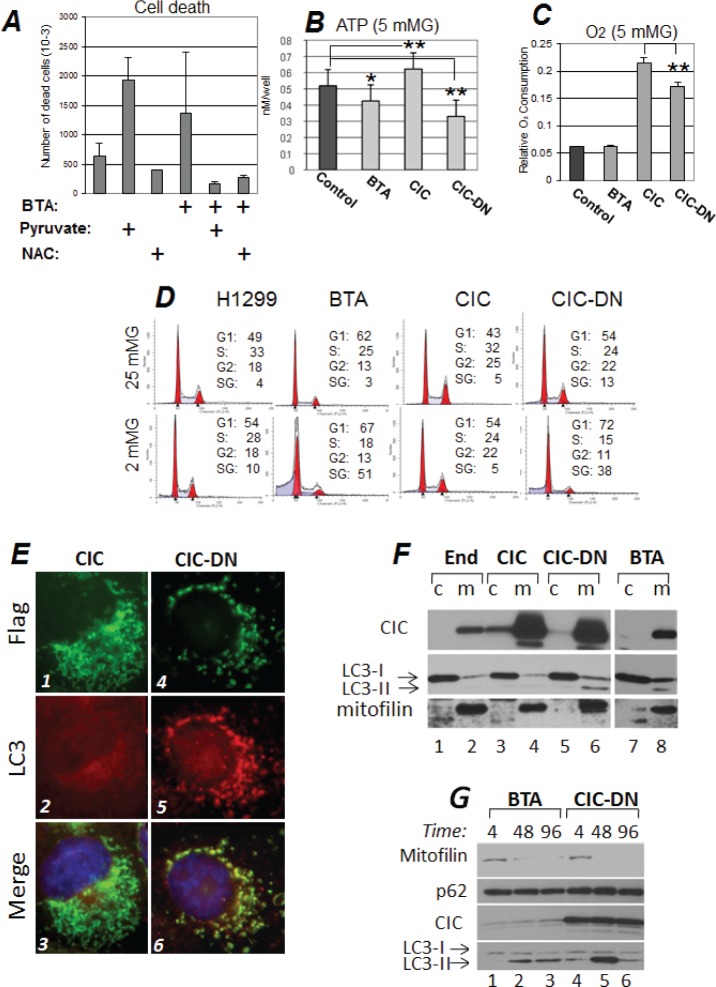
Inhibition of CIC hampers survival during metabolic stress and induces mitochondrial loss *via* autophagy A. Cell death was assessed with trypan blue exclusion in cells treated with DMSO or with BTA, in the presence or absence of 5mM methyl-pyruvate or 2 mM NAC. ATP (B) and Oxygen consumption levels (C) in cells cultured in 5 mM glucose and mock treated (DMSO, Control) or treated with BTA, or in cells expressing CIC or CIC-DN, 16 hours after treatments. D. Cell cycle profiles of control H1299 cells, or of cells treated with 1 mM BTA, or expressing the indicated CIC proteins, cultured in either 25 mM (upper panels) or in 2 mM Glucose (bottom panels). The percentage of cells distributed in the G1-, S-, or G2- phases of the cells cycle is shown. Apoptotic cells are identified as the Sub-G1 (SG) population. E. Immuno-fluorescence experiments similar to those described in Figure 4A, were performed in cells expressing epitope tagged Flag-CIC (panels 1 to 3) or Flag-CIC-DN (panels 3 to 6) proteins. Cells were stained with anti-Flag (green, panels 1 and 4) and anti-LC3 (red, panels 2 and 5) antibodies, and counterstained with DAPI. Merged images are shown in panels 3 and 6. Note the intense LC3 staining in the mitochondria of panel 5. The results of these immunofluorescence experiments where confirmed with cellular sub-fractionation experiments shown in F. Cytoplasmic (c) and mitochondrial (m) fractions derived from naïve 293T cells (lanes 1,2) or 293T cells expressing CIC (lanes 3,4) or CIC-DN (lanes 5,6) or treated with BTA (lanes 7,8), were probed with anti-CIC, anti-LC3, or anti-mitofilin antibodies, as indicated. The two arrows in the LC3 blot indicate cytosolic (-I) or activated (-II) LC3 forms. Note the enrichment of LC3-II in the mitochondria of cells expressing CIC-DN or BTA (lanes 6 and 8, respectively). All images shown are derived from the same autoradiogram at identical times of exposures, but lanes in between samples of interest were eliminated. G. Time course (in hours, indicated at the top of each panel) of BTA treated- (lanes 1 to 3) or CIC-DN induced cells (lanes 4 to 6). The anti-mitofilin, anti-p62 (employed as a general autophagic marker), anti-CIC, or anti LC3 specific immuno-blots are shown.

The collapse of MMP indicated that CIC dysfunction perturbs the proton gradient, which is directly linked to the exchange between citrate and malate promoted by CIC, as well as the flux of anaplerotic substrates across the mitochondrial membrane [1,2,13]. Specifically, the depletion of citrate induced by CIC inhibition would predictably lower the levels of malate and isocitrate, which are produced *via* citrate oxidation and isomerization, respectively. Further, the shift of metabolism towards glycolysis due to blocking of CIC activity may consume pyruvate in the cytoplasm for lactate production, thus rendering pyruvate less available for mitochondrial metabolism. Pyruvate, citrate and isocitrate can all enter the Krebs cycle at various steps generating reducing equivalents for the electron transport chain, which in turn stabilize MMP [13]. To test this hypothesis we measured the levels of several anaplerotic substrates in cells were CIC was inhibited. In support of our conjecture, the levels of malate and isocitrate were reduced (Figure 4FG), and addition to the media of pyruvate, in the form of the membrane permeable methyl-pyruvate, partially restored mitochondrial membrane polarity (Figure S5A), as well as complex I activity (not shown). Very importantly, methyl-pyruvate and NAC also partially prevented loss of viability in cells treated with BTA (Figure 5A). Thus, we argue that CIC preserves mitochondrial activity at least in part by maintaining adequate levels and flux of anaplerotic substrates, which in turn prevent depolarization of the mitochondrial membrane, while its inhibition leads to mitochondrial dysfunction. Interestingly, impairment of CIC activity also re-wires the metabolism towards glycolysis, either as a consequence of mitochondrial dysfunction (the Warburg effect) or due to the lowered concentration of citrate that enhances PFK1 activity (see scheme in Figure 1A).

### The ability of CIC to restrain the glycolytic addiction of tumor cells leads to a growth advantage

The above data indicated that CIC inhibition exacerbates the Warburg effect, a metabolic trait that is proposed to promote malignancy. At first glance this result appeared paradoxical, given that CIC is required for tumorigenesis. However, glycolysis is metabolically advantageous when glucose is abundant, because in these conditions it can generate ATP at faster rates compared to OXHOPHOS [33]. By contrast, rapidly growing tumors surpass the ability of the microenvironment to provide nutrients, and switch towards alternative metabolic pathways, including OXHOPHOS, for energy production [18]. Therefore, we hypothesized that CIC is important for adaptation when glucose is limiting. Likely due to the enhanced glycolytic behavior, at high concentrations of glucose (25 mM) oxygen consumption rates and ATP levels were not significantly modified in cells where CIC was inhibited compared to control cells (Figure S5BC). However, at lower, yet physiological glucose levels (5 mM), cells expressing CIC consumed more oxygen and produced more ATP relatively to BTA-treated or CIC-DN -expressing cells (Figure 5BC). Cell cycle analysis further revealed that glucose restriction led to induction of apoptosis in BTA treated cells, while expression of CIC, but not of CIC-DN was protective in these conditions (Figure 5D). Collectively therefore, these results provide for a model whereby CIC restrains the excessive reliance upon glycolysis allowing tumor cells to shift towards OXPHOS for ATP production, ultimately favoring survival when glucose is limiting.

### CIC is induced by mitochondrial respiration injury and prevents mitochondrial depolarization and depletion

**Figure 6:**
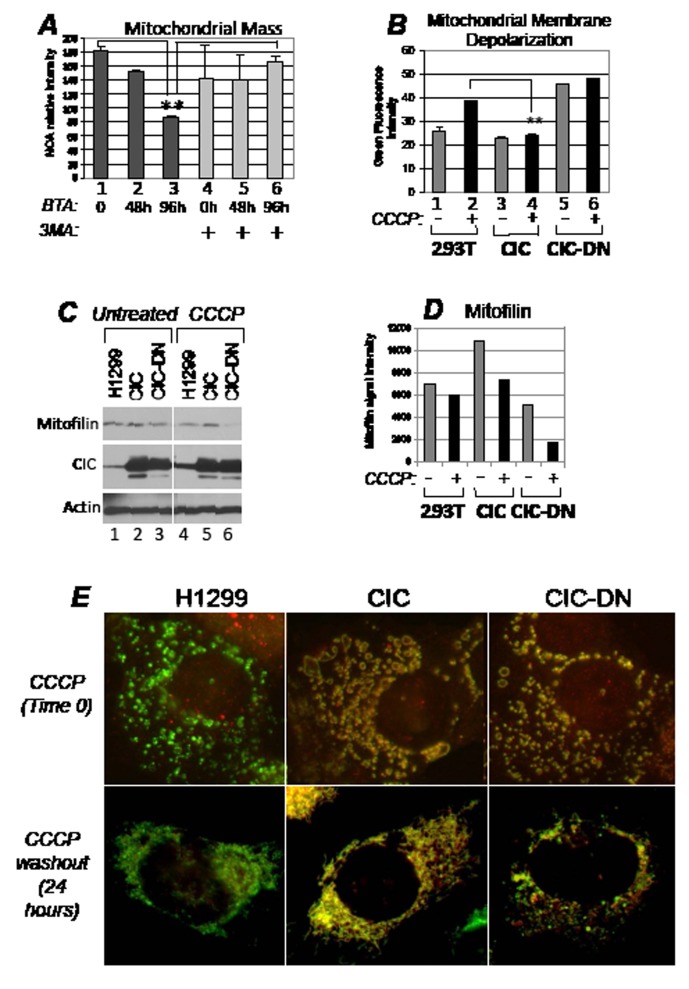
CIC protects from mitochondrial damage and depletion following respiration injury A. Assessment with NOA staining of mitochondrial mass as a function of time (in hours) in control cells (lanes 1 and 4), or in cells treated with BTA (lanes 2-3 and 5-6) and in the absence (lanes 1-to-3) or presence (lanes 4-to-6) of 5 mM 3MA. Double asterisks refer to p<0.05. Brackets refer to the comparison of values expressed in *3* versus *1*, or *3* versus *6*. B. Measurement of mitochondrial membrane depolarization with the JC1 assay in the absence (-, lanes 1,3,5) or presence (+, lanes 2,4,6) of 20 micro-M CCCP in the cell lines indicated at the bottom of each panel. The diagram shows histograms of JC1 green fluorescence intensity, indicative of depolarization, derived from a duplicate experiment. C. The H1299 cell lines indicated at the bottom of each panel were left untreated (lanes 1-3) or treated with CCCP (20 micro-M, lanes 4-6) for 16 hours, at which time CCCP was removed from the media. Cells were replenished with fresh media, incubation was carried out for additional 2 hours, after which time cell extracts were probed with the antibodies indicated at the side of each panel. Note the increase in the endogenous CIC levels in CCCP treated *versus* untreated cells (lane 4 *versus* lane 1, respectively). The image shown is derived from the same autoradiogram where lanes between the samples of interest were cropped. Panel D shows a quantification of the mitofilin signal. E. Immuno-fluorescence of H1299 cells treated with CCCP for four hours (indicated as time 0, upper panels), or after 24 hours of CCCP wash-out. All panels in this Figure show the merged signals derived from the anti-CIC (red) and anti-Hsp70 (green) staining. Approximately 70% of cells expressing CIC-DN showed the disrupted mitochondrial network even after 24 hours of recovery from CCCP, compared to control H1299 or CIC-expressing cells that displayed this phenotype in less 10% of cells.

The mitochondria of tumor cells undergo oxidative stress due to mtDNA and to oncogenic mutations that affect the activity of mitochondrial enzymes involved in respiration [21,22,23]. Mitochondrial damage in turn triggers quality control systems consisting of mitochondria degradation *via* autophagy/mitophagy [34,35]. In keeping with our previous results we suspected that CIC might exert a protective role on mitochondrial damage and disposal. First, immuno-fluorescence and cellular sub-fractionation experiments demonstrated recruitment of the autophagic machinery in the form of the active- lipidated form of LC3 and of lysosomes, to the mitochondria of cells expressing CIC-DN or treated with BTA, but not in CIC containing cells (Figure 5E, compare panels 2 and 5; Figure 5F, compare lanes 6 and 8 with lanes 2 and 4; Figure S6AB). Furthermore, kinetic studies where mitochondrial content was assessed with the marker of mitochondrial mass, mitofilin [36,37], showed a reduction of mitochondrial quantity as a function of time, following BTA treatment or CIC-DN induction (Figure 5G, compare lanes 2 and 3, and lanes 5 and 6 with lanes 1 and 4, respectively). This mitochondrial depletion coincided with activation of autophagy, as judged based on LC3 activation and conversion (Figure 5G, lower panel). Similar results were obtained when mitochondrial amount was studied with a second marker of mitochondrial content, namely Nonyl-Acridine Orange (NOA) [38] (Figure 6A, compare lanes 2 and 3 with lane 1). Blocking of autophagy with 3-Methyladenine (3MA), restored mitochondrial amount (Figure 6A, compare lanes 5 and 6 with lanes 2 and 3), thus suggesting clearance of these mitochondria by mitophagy/autophagy. Importantly, 3MA also prevented cell death induced by BTA in glucose-restricted conditions (Figure S6C).

Autophagy can be organelle selective or proceed as a bulk degradation process of multiple cellular compartments. CIC did not interfere with global autophagic flux in response to the mTOR inhibitor, rapamycin, or to glucose restriction, suggesting that it might specifically affect mitophagy (Figure S6D). To test this hypothesis further, we employed the mitochondrial respiration uncoupler carbonyl-cyanide-3-chlorophenylhydrazone, CCCP, which induces depolarization of the mitochondrial membrane and mitochondria fragmentation followed by mitophagy [i.e., 37]. Cells where CIC was abundant were protected from MMP loss induced by CCCP, while expression of CIC-DN destabilized MMP independently of CCCP and failed to restore mitochondrial membrane polarity (Figure 6B, compare lanes 3 and 4 with lanes 1 and 2; and lane 6 with lane 4, respectively). Furthermore, in conditions in which CCCP was washed out from the media after treatment, H1299 and CIC-expressing cells maintained stable mitofilin content (Figure 6C, compare lanes 4 and 5 with lanes 1 and 2; quantified in Figure 6D) as well as normal mitochondrial structure and morphology (Figure 6E). The protection seen in naïve H1299 cells correlated with an induction of the expression levels of endogenous CIC by CCCP (Figure 6C, compare lane 1 *versus* lane 4 in the CIC immuno-blot). Other types of mitochondrial stressors, such as rotenone, also induced CIC expression levels (Figure S6E).

Viewed as a whole, these results indicate that CIC is a sensor and an effector of mitochondrial stress. *Via* its citrate export ability, CIC stabilizes membrane potential minimizing mitochondrial depletion. By contrast, cells where CIC is inhibited trigger autophagic clearance of mitochondria.

### The knock-down of the CIC orthologous gene in *zebrafish* induces mitochondrial loss and activation of autophagy

**Figure 7:**
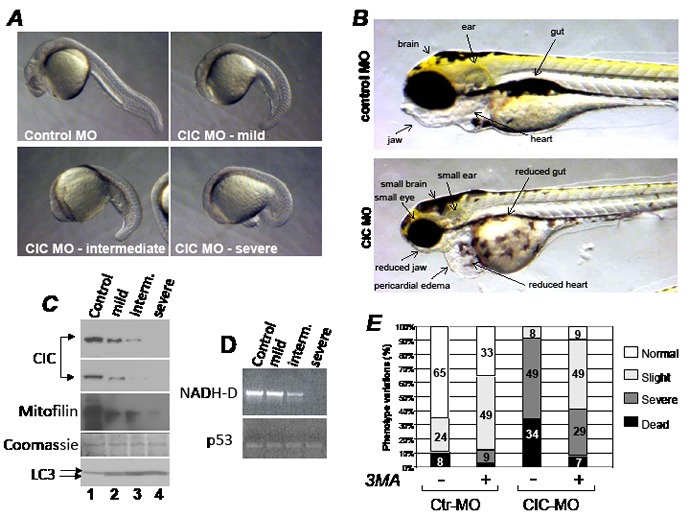
The knock-down of CIC in *zebrafish* results in mitochondrial DNA depletion and activation of autophagy A. Zebrafish embryos injected with control morpholino (Control MO) or with three different doses of the CIC specific morpholino (CIC MO), resulting in a progressively abnormal phenotypes classified from mild to severe. B. Phenotypes of zebrafish injected with control or CIC MO at 4 days post-development. Images were captured at identical magnification. C. Immuno-blot of pools of 15-30 embryos of each phenotype shown in A, with antibodies listed at the side of each panel. Two different exposures of the CIC-specific immuno-blot are shown. Like in human cell extract, in zebrafish extracts the two main LC3 forms, I and II, could be detected and are indicated by arrows. D. DNA extracted from pooled embryos representative of each phenotype was probed in semi-quantitative PCR by using primers that amplify either 1Kb of mtDNA encompassing the mitochondrial gene NADH-dehydrogenase (NADH-D; top panel), or the nuclear p53 gene (bottom panel). E. Survival of embryos injected with control MO or with CIC-MO grown in media containing (+) or lacking (-) 5 mM 3MA. The percentages of each phenotype classified as normal, slight, severe and dead are indicated by bars of different colors.

Genes that promote tumorigenesis are often necessary for embryonic development, and some of these genes are metabolic modulators, given that embryogenesis and tumorigenesis are energetically demanding [39,40]. Additionally, in keeping with the hypothesized involvement of CIC in chromosome 22q11.2 microdeletion syndromes in humans [5], we deemed important to investigate whether changes of CIC dosage affects embryogenesis in the model organism *zebrafish*. This approach was possible because human and *zebrafis*h CIC proteins display a high degree of homology (Figure S7). By using a morpholino-based (MO) knock-down strategy, we found that the CIC MO led to a stark dose-dependent phenotype, and produced a relatively small percentage of dead embryos (34%, Figure 7A, Figure 7E). At 24 hours post-fertilization the head was flattened and reduced along the ventral-dorsal axis and at later steps of development CIC morphants displayed a prominent reduction of the entire cranial region, involving the size of the brain and the jaw. The heart was small and surrounded by pericardial edema, indicative of cardiac dysfunction (Figure 7B). Confirming the efficiency of the knockdown, a progressive decline of the expression levels of CIC was seen with increasing doses of MO, paralleled by a reduction in the levels of mitofilin and of mitochondrial, but not nuclear DNA (Figure 7C and Figure 7D, respectively). Similarly to what observed in human tumor cells, LC3 total levels and the conversion to the lipidated form were starkly increased (Figure 7C, compare lanes 2 to 4 with lane 1 in the LC3 immunoblot), and the phenotypes of the CIC-MO were largely rescued by 3MA (Figure 7D). Noticeably, CIC morphants recapitulated features of VCFS, which is characterized by multiple abnormalities including cardiac defects, facial and jaw anomalies and cleft palate [41]. The effects of CIC on the development of the brain as well as on the mitochondria could also explain the delay in cognitive functions and the learning disabilities that occur in some forms of this disease. Together with other gene products within chromosome 22q11.2, CIC deficiency might therefore contribute to- or act as a modifier of- the complex spectrum of pathological features occurring in human velo-cardio-facial syndromes.

## DISCUSSION

**Figure 8:**
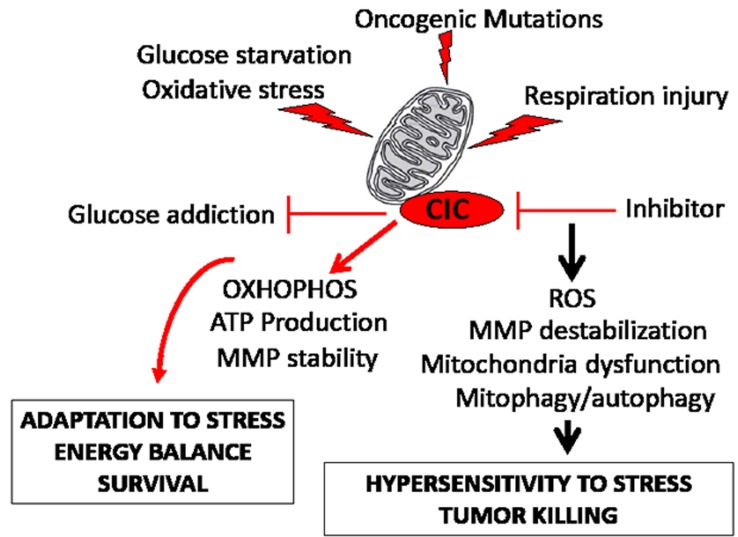
CIC maintains mitochondrial homeostasis in tumor cells The diagram summarizes the findings (see also the Discussion). The mitochondria of tumor cells endure oxidative and respiration stress caused by the nutrient restricted microenvironment, by oncogenic activation, and by altered activity of mitochondrial enzymes. We have shown here that high CIC levels in tumors allows for adaptation to nutritional stress and resistance to mitochondria respiration injury. This protective effect of CIC on the tumor mitochondria relies upon an inhibition of glycolysis, promotion of mitochondrial OXOPHOS and ATP production, and stabilization of the mitochondrial membrane potential (MMP). We have mechanistically linked these activities of CIC to its ability to promote the export of citrate and to maintain adequate levels and flux of other anaplerotic substrates. By contrast, genetic or pharmacologic inhibition of CIC increases ROS levels, compromises respiratory capacity and leads to mitochondrial dysfunction and depletion *via* autophagy, preventing adaptation to oxidative or respiration stress, and ultimately leading to tumor killing.

In this study, we have conducted, for the first time, a functional characterization of the mitochondrial transporter CIC, a gene product highly conserved throughout evolution from *yeast* to humans. Our findings have identified CIC as an important determinant of the homeostatic control of tumor mitochondria, through which activity CIC becomes essential to the cancer promoting metabolic program (depicted in Figure 8). We have shown that high CIC levels in tumors preserve a critical threshold of mitochondrial activity and amount that allows adaptation during metabolic and respiration stress. Importantly, embryonic tissues and tumor cells appear selectively sensitive to CIC inhibition compared to adult normal tissues. This sensitivity may be due to the already high extent of oxidative damage, of ROS, and of mitochondrial stress due to differentiation programs during development [42] and to oncogenic signal pathways in tumors respectively, which could then reach levels incompatible with proliferation when CIC is inhibited. Importantly, new evidence now demonstrates the synthetic lethal activity of compounds that increase ROS production in various tumors [23]. These agents achieve tumor killing with a remarkable degree of selectivity.

Based on these considerations, we believe that the CIC inhibitor compound, BTA, provides a reasonable platform for the design of a new class of agents that target mitochondrial metabolism and turnover. There are currently few examples of drugs that exploit the intrinsic vulnerability of tumor mitochondria to achieve selective cancer killing [43] and the development of such agents might improve therapeutic responses [44]. Given the inability of cells where CIC is dysfunctional to tolerate nutrient restriction, it can also be predicted that CIC inhibitors might act synergistically with drugs that interfere with glucose metabolism, for example with 2-deoxyglucose or metformin, that are currently being evaluated in cancer therapy [45]. Therefore, our results should provide a rationale for testing of CIC inhibitors, alone or in combination with other metabolic modulators, as anti-cancer agents.

Albeit the fact that CIC inhibition exacerbates the Warburg effect and yet has anti-tumor activity may appear paradoxical, there are several explanations for this observation. Based on our findings, we propose that CIC functions to balance the potential detrimental effects that arise when the excessive dependence upon glycolysis of tumor cells overpowers the ability of the environment to adequately supply nutrients, and/or if the extent of oxidative stress endured by tumor cells reaches levels such to trigger excessive degradation *via* mitophagy [46]. However, given the incredible plasticity of tumor cells and their ability to adapt to metabolic pressure, it will be very important to interrogate the long term effects of the anti-tumor activity of BTA in many other tumor types of different tissue origin as well as in tumor prone animal models.

Based on data presented here and available in literature, it further appears reasonable to predict the involvement of CIC in other human pathogenic conditions hallmarked by mitochondrial oxidative damage and by alterations of mitochondrial turnover. Aside from chromosome 22q11.21 microdeletion syndromes that alter mitochondrial plasticity [4], mitochondrial homeostasis is perturbed in human disease states ranging from diabetes, neurodegeneration and aging. Interestingly, an analysis of existing gene expression databases derived from Parkinson's disease, shows statistically significant alterations of CIC expression levels in various regions of the brain of affected patients relatively to normal healthy control individuals (http://www2.cancer.ucl.ac.uk/Parkinson_Db2/php/insert_symbol.php?region=1 and region=1 and region=1 and region=1 and choice=slist and list=SLC25A1 and Pvalue=0.05 and LogFC= and NegLogFC= and AdjPvalue=). A common denominator of neurodegeneration, aging and cancer consists in alterations of mitochondrial activity and turnover [47, 48]. Importantly, in the fruit fly a member of the tricarboxylate transporter family, *INDY*, promotes longevity [10]. Thus, in addition to being detrimental to tumor growth, CIC inhibition might -perhaps advantageously- interfere with other life-shortening conditions. Based on these considerations, it is possible that CIC is central to many pathways linked to human health and thus, targeting of its activity, positively or negatively, could be beneficial to various diseases.

## METHODS

### Cells, reagents, antibodies, primers

The cell lines employed in this study were obtained from the tissue culture core facility at LCCC. H1299 cells were obtained from ATCC. Cells were grown in Dulbecco's modified Eagle's medium (DMEM, 5 mM glucose, with glutamine and pyruvate from Invitrogen) and supplemented with 10% fetal calf serum (FCS). Early passage cell lines or frozen pellets were used for screening of CIC expression levels (Fig.S1 and Fig.1A). The normal and tumor breast cancer specimens employed for CIC analysis were obtained from the Histopathology and Tissue Shared Resource, Georgetown University Hospital. All the reagents for the study of complex I activity were purchased from Sigma-Aldrich and were as follows. Succinate: #S3674-100G; KCN: #60178-25G; Coenzyme Q1: #C7956-10MG; Decylubiquinone: #D7911; 2,6-dichlorophenol-indophenol (DICP):# D1878-5G; Thenoyltrifluoroacetone: 88300-5G; rotenone: #R8875; NADH: #N4505. 1,2,3, Benzenetricarboxylica acid (BTA), was from Sigma (# B4201). The CIC specific shRNA vectors were purchased from Origene (#TG316728 and #TR316728, untagged and GFP-tagged). The vectors expressing human CIC untagged or Flag-Myc epitope tagged were also from Origene (#SC120727 and RC200657, respectively). The antibodies used in this study were as follows. The anti-CIC antibody from Santa Cruz Biotech, # sc-86392 employed at 1:1000 dilution in immuno-blot and at 1:100 in immuno-fluorescence; the anti-mHsp70 and anti-mitofilin antibody were from Novus Biological (#NB300-527 and #NB100-1919, respectively) employed at 1:500 in immuno-fluorescence and 1:1000 in immuno-blot; for the LC3 immuno-blot a mixture of two antibodies was used each at 1:1000 dilution (LC3 MBL #PM036; LC3 Novus Biol. # NB100-2220). The LAMP-1 antibody was from Abcam (#H4A3). The sequences of primers used were: the human cytochome c oxidase: forward: 5'-TCGCCGACCGTTGACTATTCTCT-3'; Reverse:5′ AAGATTATTACAAATGCATGGGC-3′. The zebrafish NADH-dehydrogenase: forward: 5'-AGGGTTGCTGGGATGGCAGAGCTCGGTA-3'; reverse:5' CACTCCATCAAAGTGACCCCTTAGCAT-3'. The zebrafish p53: forward: 5'-ACATGA AATTGCCAGAGTATGTGTC-3'; Reverse: 5' TCGGATAGCCTAGTGCGAGC 3'.

### Strategy for the generation of CIC wild-type and CIC mutant expressing vectors, and of stable cell lines

The two cDNA clones expressing human untagged CIC and epitope tagged CIC were cloned into the pcDNA4/TO tetracycline regulated vector (Santa Cruz, T-Rex system). Stable tetracycline inducible cell lines were obtained as described previously.

### Quantification of metabolites, ATP, oxygen, ROS, MMP and mitochondrial mass

The concentration of citrate, lactate, isocitrate, and malate were assessed using specific kits from BioVision. Cellular ATP levels were determined via the ATP kit (Promega), while oxygen consumption rates were measured using the BD oxygen biosensor systems (OBS) from BD Bioscience. Triplicate samples of 50,000 cells were seeded onto 96-well OBS plates. The number of cells was determined at each time point by sampling cells seeded into side-by-side plates. Fluorescence was measured from the bottom of the well every 24 h and measurements were normalized by subtracting the reading from the same well prior to the addition of the cells (blank). For measurement of ROS we used H2DCFDA (Invitrogen). Mitochondrial mass was assessed by using mitotracker green (Invitrogen), as per manufacturer instructions, or Nonyl Acridine Orange at a final concentration of 300 nM, followed by analysis by flow cytometry. Mitochondrial Membrane Potential (MMP) was studied with the JC1 assay kit from Cayman.

### Mice and tumors

To produce tumor xenografts 5 × 10^6^ cells were resuspended in PBS and injected subcutaneously in the flanks of female nude mice. For drug treatments mice were randomized to receive either PBS or a PBS solution of BTA at a concentration 26 mg/kg which was administered via intra-peritoneal route three times a week. Mice were pre-treated twice prior to the inoculation of tumor cell lines. Once detectable tumors started to form, their size was measured with a caliper in three dimensions. Serial measurements were made at two-three day intervals after the identification of the initial cellular mass to determine growth curves *in vivo*. Tumor volume was calculated using the formula for a prolate spheroid: volume = (4/3) × *a*^2^*b*, where *a* is the width and *b* is the length. All animals were sacrificed when the tumors exceeded 1.5 cm. At the completion of experiments, tumors were excised, weighed and statistical significance of differences in tumor volume were made using two factor repeated measures analysis of variance followed by Fisher's last significant difference test for multiple comparisons. The trial administration of BTA to explore its toxic effects, was conducted on 8 non immuno-compromized mice, randomized in two groups and injected as described before for five consecutive months. Animals were monitored once a week for the presence of signs of disease, particularly neurological disturbances or weight loss, and they were weighted periodically. All animal studies were approved by the Georgetown University Institutional Animal Care and Use Committee.

### Statistical analysis

Data are expressed as means ± standard deviations (SD). The two-tailed Student *t* test was used for all statistical analysis of experiments presented and Excel was used for statistical calculations. Significant differences are indicated using the standard Michelin Guide scale (*P* < 0.05, significant; *P* < 0.01, highly significant; *P* < 0.001, extremely significant).

Additional materials and methods are provided as Supplementary information.

## Supplementary Figures


