# Supplementary material for: The mitochondrial citrate transporter, CIC, is essential for mitochondrial homeostasis
Source: Oncotarget. 2012 Oct 20;3(10):1220–35. doi: 10.18632/oncotarget.714 (PMC3717962; doi:10.18632/oncotarget.714)
Supplement: Supplementary file 1 [file oncotarget-03-1220-s001.pdf]

# The mitochondrial citrate transporter, CIC, is essential for mitochondrial homeostasis - Catalina-Rodriguez et al

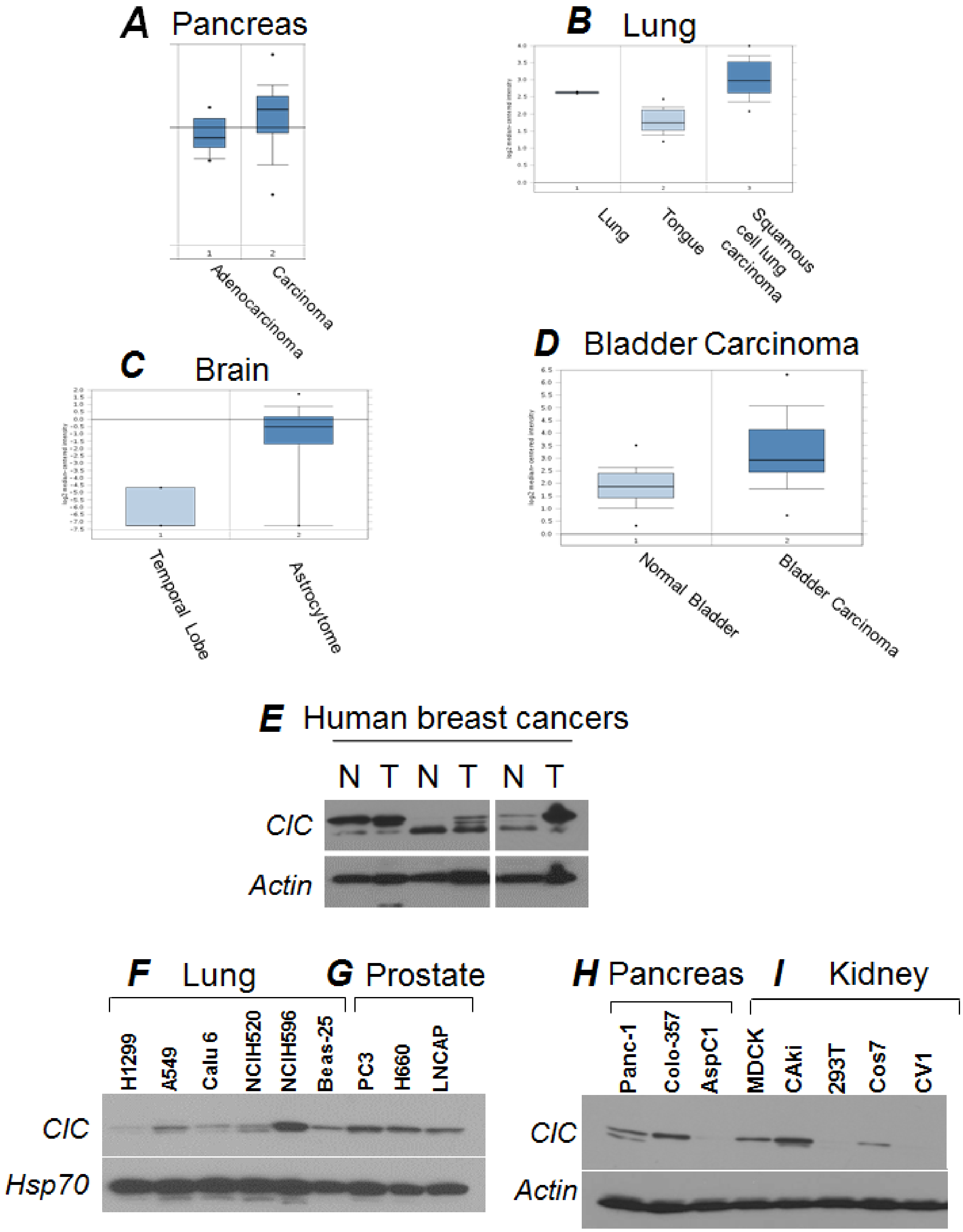


**Figure S1: CIC levels are increased in human tumors.** A-D. CIC mRNA expression levels in human normal or tumor tissues indicated at the top of each panel. Data were obtained from the Oncomine database [49] by selecting a minimal threshold p-value of 0.0005. E. CIC expression levels in three human breast carcinomas. Total cell extracts derived from normal tissues (N) or tumor samples (T) were probed with anti-CIC or anti-actin antibodies. F-I. Analysis of CIC expression levels in human cell lines and some NCI-60 cells, at the top of each panel.

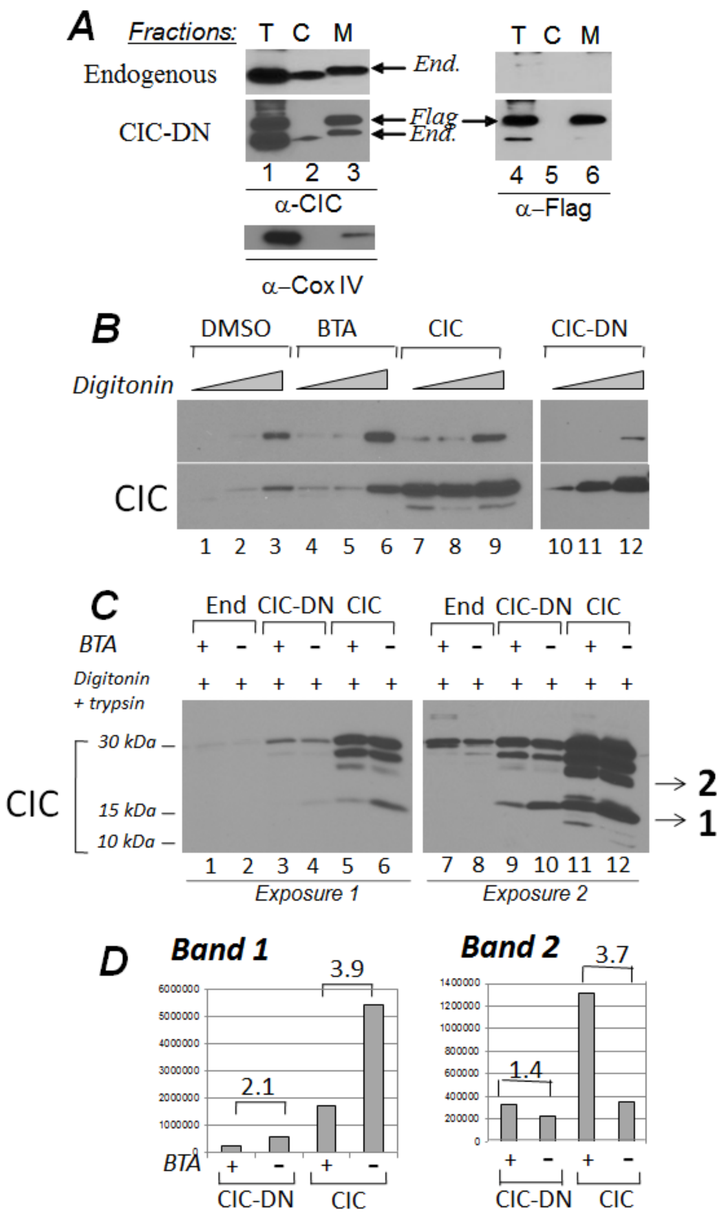


**Figure S2: Characterization of the CIC dominant negative (CIC-DN) mutant.** A-B. CIC-DN does not disrupt sub-cellular localization and colocalizes with endogenous CIC. A. 293T cells transfected with the pcDNA4/TO vector or with the vector expressing a epitope-flag-CIC-DN were fractionated to obtain cytosolic (indicated as *C*, lanes 2 and 5), or mitochondrial (*M*, lanes 3 and 6) fractions. Total lysates derived from the same number of cells were prepared in parallel (T, lanes 1 and 4). Extracts were probed with anti-CIC-, anti Flag- or anti-COX IV antibodies. Arrows indicate the position of the endogenous (indicated as *End*), or of the expressed-CIC protein (indicated as *Flag*). B. CIC-DN does not affect the ability of CIC to embed in the micothondial membrane. Mitochondria sub-fractionationation experiments were performed in 293T cells containing pcDNA4/TO vector, and treated with DMSO (lanes 1-3), or with 1 mM BTA (lanes 4-6), or in the tetracycline inducible cells harboring CIC (lanes 7-9), or CIC-DN (lanes 10-12). Approximately 100 micrograms of isolated mitochondria (normalized with a Bradford assay), were divided into three aliquots per treatment sample, each of which received digitonin at a concentration of 0.2% (lanes 1, 4, 7,10), or 0.4% (lanes 2,5,8,11) or 1% (lanes 3,6,9,12). After incubation for 30 minutes at 37 $^{o}$C, mitochondria were removed by centrifugation and the supernatants were probed in immuno-blot with anti-CIC antibodies. Mitofilin was used as a reference for inner mitochondrial membrane extraction. C. Impaired reactivity of CIC-DN with BTA. Mitochondria isolated from control cells (lanes 1,2) or from cells expressing CIC-DN (lanes 3,4) or CIC (lanes 5,6) were incubated with 2 mM BTA (lanes 1, 3 and 5) or DMSO (lanes 2,4 and 6) for 10 minutes at room temperature, followed by incubation with digitonin (0.4%) and trypsin (20 mg/ml). Reactions were then probed in immuno-blot with the anti-CIC antibody (Santa Cruz) that recognizes an epitope comprised between amino acids 225 and 275, therefore nearby the residues targeted for mutagenesis in the CIC-DN protein. Arrows indicate two fragments that are protected from tryptic digestion in the presence of BTA. Two different exposures of the same autoradiogram are shown. D. Signal intensities of bands *1* and *2* at comparable exposures derived from the autoradiograms shown in *C* were calculated with the ImageJ software (NIH), and plotted in the shown diagram. Brackets and numbers at the top of the histograms refer to fold differences of band 1 and 2 intensities in CIC or CIC-DN expressing cells.

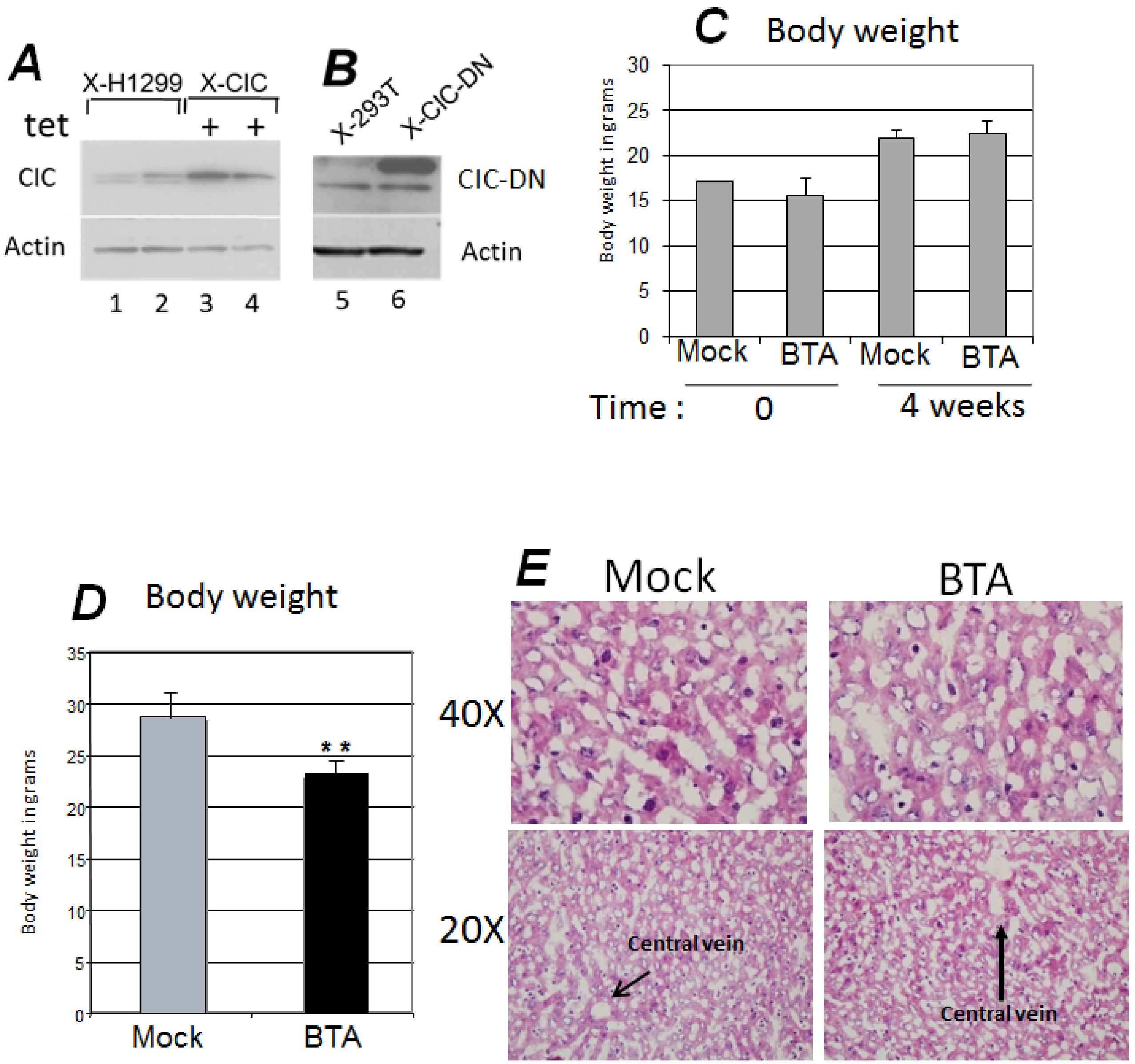


**Figure S3: Effects of CIC inhibition and of BTA treatment *in vivo*.** A-B. Total cell lysates from tumors derived from the injection experiments described in Fig. 2 A and B were probed in immuno-blot with anti-CIC, anti-actin antibodies. Two tumors of H1299-CIC cells without (lanes 1 and 2) or with tetracycline (lanes 3 and 4), are shown A. Tumor extracts of control- or CIC-DN- containing cells are shown in B. C. Body weights of immuno-deficient mice injected with MBA-MD-231 cells and randomized in two groups (mock and BTA treated). Animals were weighted before injections (time 0) and at completion of the experiment (4 weeks after). D-E. A trial administration of BTA for a period of five months in mice is well tolerated. D. Eight non immuno-compromised mice were randomized in two groups, as mock and BTA-treated animals. BTA was administered via intra-peritoneal route, twice or three times a week at a dose of 26mg/Kg, for a period of five consecutive months. Animals were weighted at completion of the trial. There was a 18% reduction of body weight in the BTA treated animals, which however did not impact upon survival, as all animals survived the experiment. E. H&E stained sections of the liver at 40X and 20X magnification. The liver was chosen for this analysis, because CIC is highly expressed therein, and plays an important role in liver metabolism.

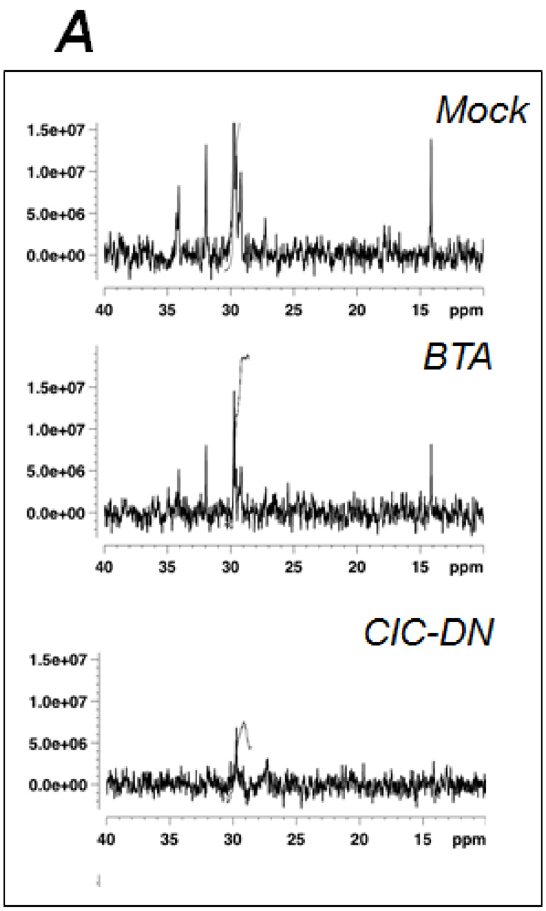


B

| | Mock | BTA | CIC-DN |
|---|---|---|---|
| FA -(CH2) | 100% | 28% | 17% |
| Total FA | 100% | 75% | 82% |
| Lactate C3 | 100% | 174% | 80% |

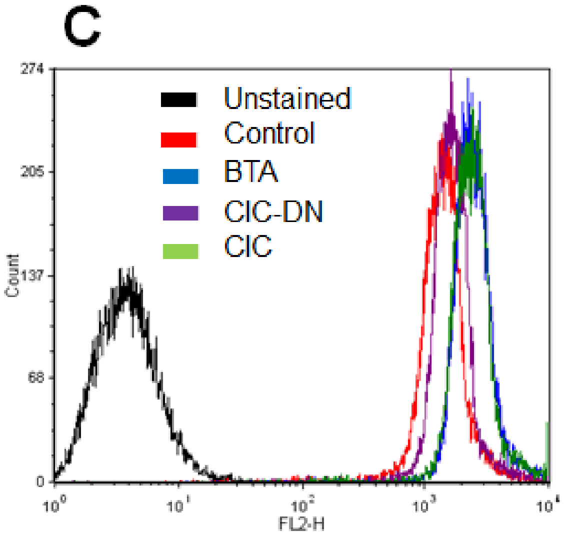


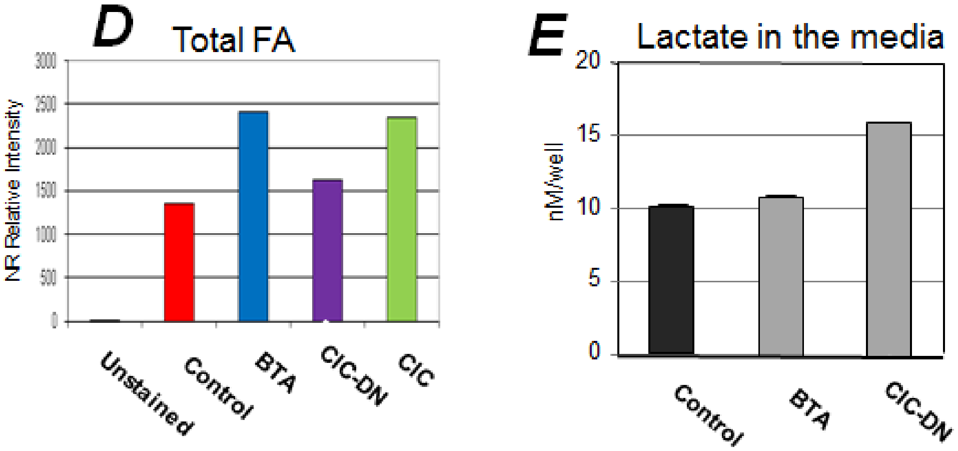


**Figure S4: CIC inhibition reduces glucose conversion to fatty acids, but it does not affect the total lipid pool.** The metabolism of D-[1,6-$^{13}C_2$] glucose added in the culture media of 293T cells treated with DMSO (mock, top panel), or treated with 1 mM BTA (middle panel) or expressing CIC-DN (bottom panel), was tracked by $^{13}$C NMR. A. Partial $^{13}$C NMR spectra of the extracted lipids. B. Differences in metabolites in 293T cells. *De novo* synthesis of fatty acids (FA), expressed as % FA –$(CH2)_n$– , was estimated from the integration of the $^{13}$C NMR signals from the extracted lipids. The steady state (total FA) was derived from the $^{1}$H NMR signals from the same samples. The intracellular lactate levels were estimated from the integrated lactate C-3 signals in the 13C NMR spectra from the aqueous phases of the extracts. All the values are normalized to control (mock). C-D. The total levels of lipids were assessed with Nile Red (NR) staining and flow cytometry. Unstained cells or cells treated as described at the top of panel C, were incubated with 1 mM Nile Red for 15 minutes before being analyzed. E. Lactate concentration (expressed in nM/well) in the tissue culture media of cells treated with DMSO, 1mM BTA, or expressing CIC-DN, respectively.

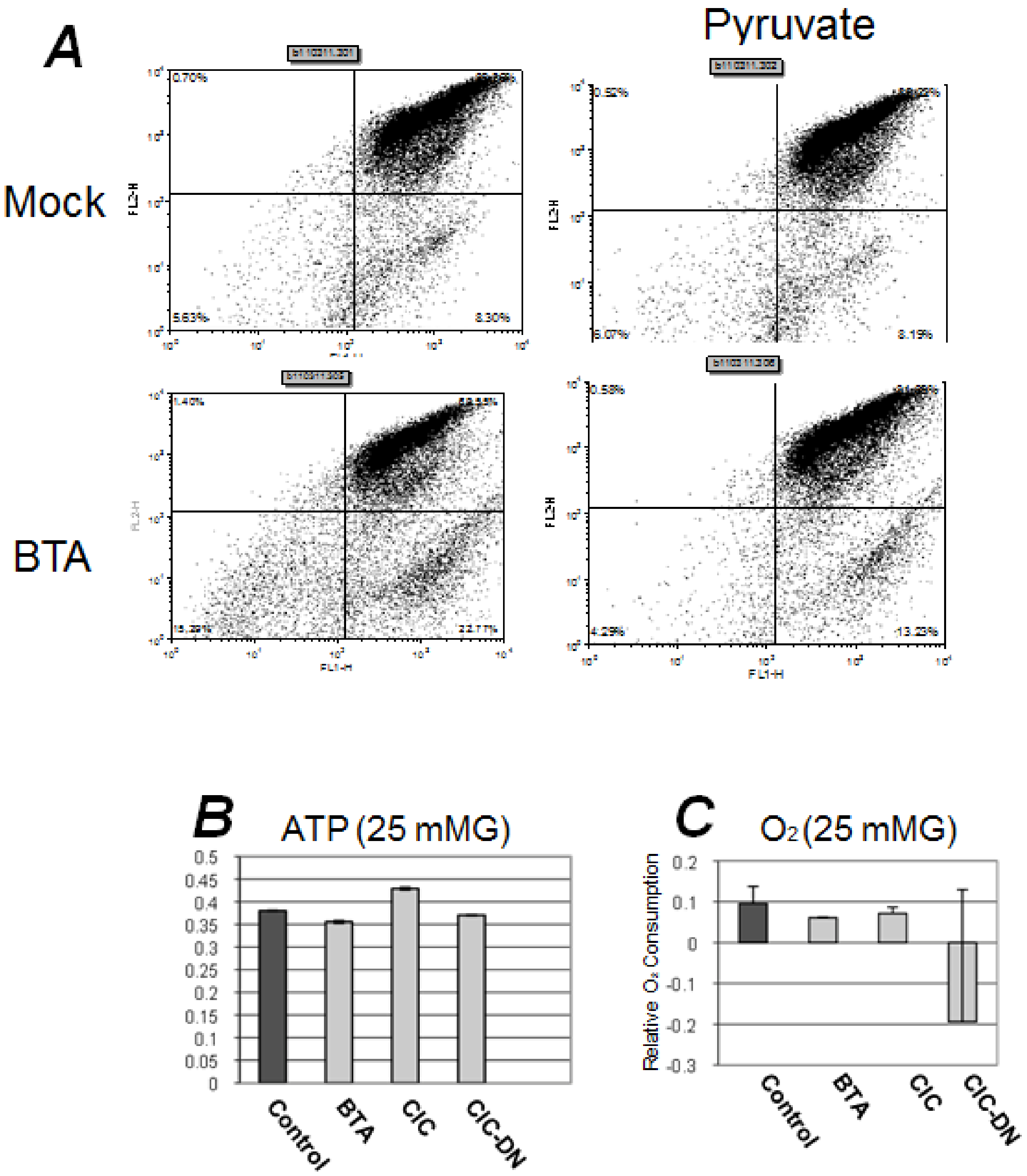


**Figure S5: A.** Pyruvate rescues MMP loss in BTA treated cells. The diagram shows a JC1 assay in 293T cells treated with DMSO or with BTA in the presence or absence of 5 mM methyl-pyruvate. B-C. ATP and oxygen consumption levels in cells treated with DMSO (control), or with BTA, or expressing CIC or CIC-DN cultured in 25 mM glucose. Error bars refer to standard deviations and negative values, when present, are due to subtraction of background values.

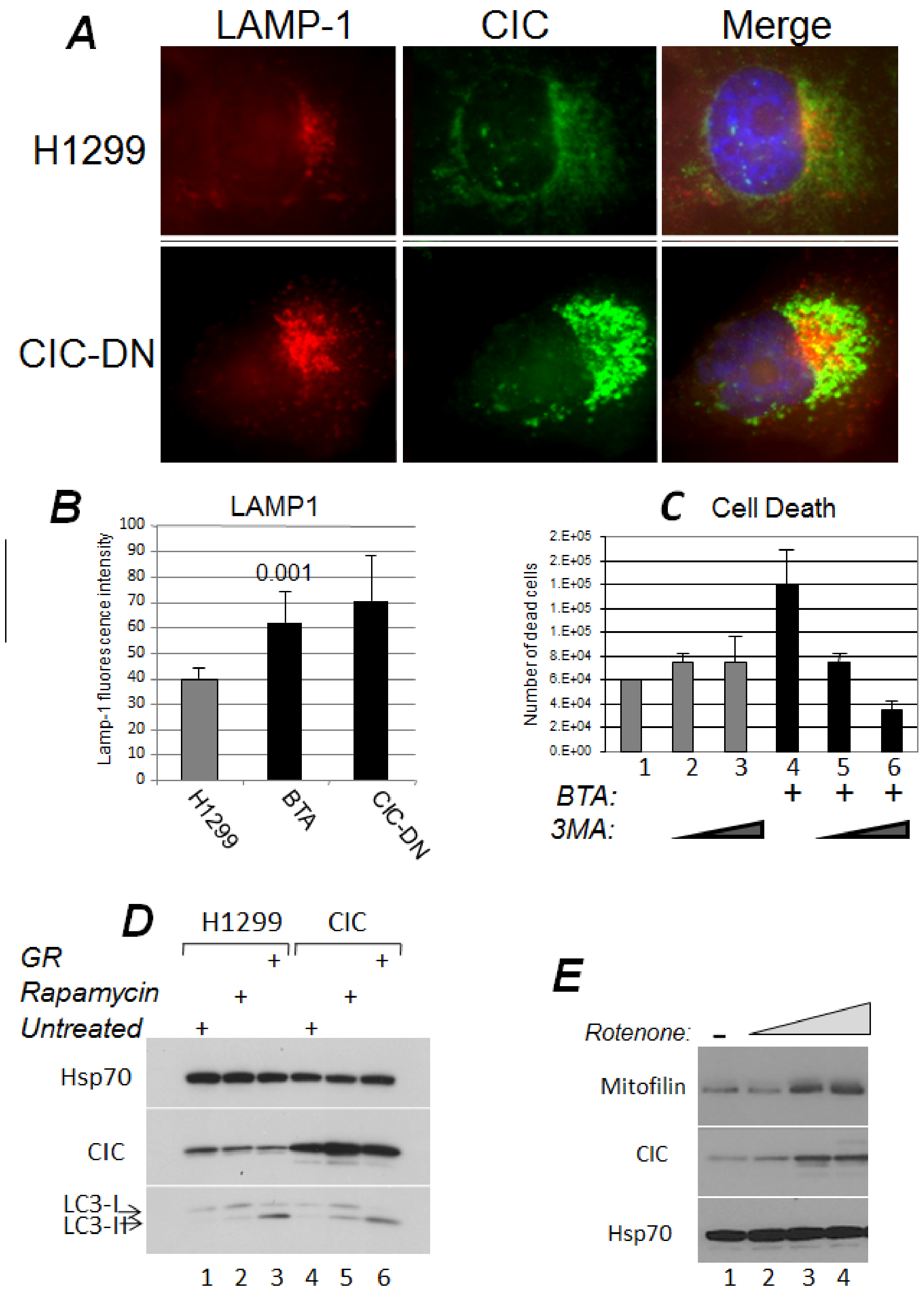


**Figure S6: Effects of CIC on mitophagy/autophagy.** A. Control H1299 cells or cells expressing CIC-DN were grown on glass coverslips for 48 hours in the presence of tetracycline. Cells were stained with antibodies specific for CIC (green), or for the lysosomal marker LAMP1 (red), and counterstained with DAPI. The DAPI signal is shown only in the merged images. B. Quantification of the LAMP1 fluorescence intensity, from H1299 cells, H1299 treated with 1 mM BTA or CIC-DN cells. Immuno-fluorescence was performed as described in A, and the intensity of LAMP1 signal was calculated with the ImageJ software. Values derived from different fields were plotted in the histograms shown. C. Effects of 2.5 mM (lanes 2 and 5) or 5 mM (lanes 3 and 6) 3MA, in untreated (lanes 1-to-3) versus BTA treated cells (lanes 4-to-6) cultured in the presence of 2.5 mM Glucose. Cell viability was assessed with trypan blue exclusion. D. H1299 cells (lanes 1-to-3) or cells expressing CIC (4-to-6) were treated with rapamycin (20 microgrograms/ml) (lanes 2 and 4) or cultured in media lacking glucose (GR, lanes 3 and 6). Cell extracts were prepared and probed in immuno-blot with anti-CIC, anti-LC3 or anti-Hsp70 antibodies. The two forms of LC3, soluble and autophagosome-associated, are indicated by arrows. E. CIC is a sensor of mitochondrial respiration injury. H1299 cells were treated with vehicle control (DMSO) (-), or with 100, 200 or 500 ng/ml of rotenone for three hours. Cell extracts were prepared and probed with the antibodies indicated at the side of each panel. Note the increase in CIC expression at higher concentrations of rotenone (lanes 3 and 4).

*Danio Rerio CIC : GenBank: AAI54413.1*
*Human CIC : GenBank: AAB08515.1*
*Identities = 253/302 (84%), Positives = 283/302 (94%), Gaps = 1/302 (0%)*

```
Human      CIC

Zebrafish  CIC   1    REQETLSCQNTSRARHFTYDHRLHNLHHFCCWFVRRPRFKTRKMSGSPKFVSPFHR        56

Human CIC        7    PRALAAAAPASGKAKLTHPEKAILAGGLAGGIEICITFPTEYVKTQLQLDERSHPPRYRG    66
                      P    L+AAAPA  G+AKLTHP   KAILAGG+AGGIEICITFPTEYVKTQLQLDE+++PPRY+G
Zebrafish  CIC  57    PHCLSAAAPA-GQAKLTHPGKAILAGGIAGGIEICITFPTEYVKTQLQLDEKANPPRYKG    115

Human CIC       67    IGDCVRQTVRSHGVLGLYRGLSSLLYGSIPKAAVRFGMFEFLSNHMRDAQGRLDSTRGLL    126
                      I  DCV+QTV+   HGV  GLYRGLSSLLYGSIPKAAVRFG +FEFLSN  MRD      G+LDSTRGL+
Zebrafish  CIC 175    IVDCVKQTVQGHGVKGLYRGLSSLLYGSIPKAAVRFGVFEFLSNQMRDES GKLDSTRGLI  175

Human CIC      127    CGLGAGVAEAVVVVCPMET I KVKFIHDQTSPNPKYRGFFHGVREIVREQGLKGTYQGLTA  186
                      CGLGAGVAEAVVVVCPMET+KVKFIHDQTS   NPKYRGFFHGVR  IVR  QGLKGTYQGLTA
Zebrafish  CIC 176    CGLGAGVAEAVVVVCPMETVKVKFIHDQTSANPKYRGFFHGVRGIVRTQGLKGTYQGLTA  235

                                                              CIC antibody epitope
Human CIC      187    TVLKQGSNQAIRFFVMTSLRNWYRGDNPNKPMNPLITGVFGAIAGAASVFGNTPLDVIKT    246
                      TVLKQGSNQAIRF +VMT+LRNWY+GDNPNK   + NP+ TG+FGA+AGAASVFGNTPLDVIKT
Zebrafish  CIC 236    TVLKQGSNQAIRFYVMTALRNWYKGDNPNKSINPVVTGLFGAVAGAASVFGNTPLDVIKT    295

                               CIC antibody epitope
Human CIC      247    RMQGLEAHKYRNTWDCGLQILKKEGLKAFYKGTVPRLGRVCLDVAIVFVIYDEVVKLLNK    306
                      RMQGLEAHKY++T    DC    ++I +KEG     AFYKGTVPRLGRVC +DVAIVF+IY+EVVK+LNK
Zebrafish  CIC 296    RMQGLEAHKYKS TV DCAIKIMKYEGPAAFYKGTVPRLGRVCMDVAIVFIIYEEVVKVLNK  355

Human CIC      306    VW                                                              308
                      VW
Zebrafish  CIC 356    VW                                                              357
```

**Figure S7: Alignment of the protein sequences of human and zebrafish CIC proteins.** The genebank accession numbers are shown at the top of the Figure. The region outlined in bold contains amino acid residues recognized by the anti-CIC antibody employed in the immuno-blot experiments (Santa Cruz, see materials and methods).

# SUPPLEMENTARY MATERIALS AND METHODS.

## Cells, reagents, antibodies, primers.

The cell lines employed in this study were obtained from the tissue culture core facility at LCCC. H1299 cells were obtained from ATCC. Cells were grown in Dulbecco' s modified Eagle's medium (DMEM, 5 mM glucose, with glutamine and pyruvate from Invitrogen) and supplemented with 10% fetal calf serum (FCS). Early passage cell lines or frozen pellets were used for screening of CIC expression levels (Fig.S1 and Fig.1A). The normal and tumor breast cancer specimens employed for CIC analysis were obtained from the Histopathology and Tissue Shared Resource, Georgetown University Hospital. All the reagents for the study of complex I and II activity were purchased from Sigma-Aldrich and were as follows. Succinate: #S3674-100G; KCN: #60178-25G; Coenzyme Q1: #C7956-10MG; Decylubiquinone: #D7911; 2,6-dichlorophenol-indophenol (DICP):# D1878-5G; Thenoyltrifluoroacetone: 88300-5G; rotenone: #R8875; NADH: #N4505. 1,2,3, Benzenetricarboxylica acid (BTA), was from Sigma (# B4201). The CIC specific shRNA vectors were purchased from Origene (#TG316728 and #TR316728, untagged and GFP-tagged). The vectors expressing human CIC untagged or Flag-Myc epitope tagged were also from Origene (#SC120727 and RC200657, respectively). The antibodies used in this study were as follows. The anti-CIC antibody from Santa Cruz Biotech, # sc-86392 employed at 1:1000 dilution in immuno-blot and at 1:100 in immuno-fluorescence; the anti-mHsp70 and anti-mitofilin antibody were from Novus Biological (#NB300-527 and #NB100-1919, respectively) employed at 1:500 in immuno-fluorescence and 1:1000 in immuno-blot; for the LC3 immuno-blot a mixture of two antibodies was used each at 1:1000 dilution (LC3 MBL #PM036; LC3 Novus Biol. # NB100-2220). The LAMP-1 antibody was from Abcam (#H4A3). The sequences of primers used were: the human cytochome c oxidase: forward: 5'-TCGCCGACCGTTGACTATTCTCT-3'; Reverse:5′ AAGATTATTACAAATGCATGGGC-3′. The zebrafish NADH-dehydrogenase: forward: 5'-AGGGTTGCTGGGATGGCAGAGCTCGGTA-3'; reverse:5' CACTCCATCAAAGTGACCCCTTAGCAT-3'. The zebrafish p53: forward: 5'-ACATGA AATTGCCAGAGTATGTGTC-3'; Reverse : 5' TCGGATAGCCTAGTGCGAGC 3'.

## Strategy for the generation of CIC wild-type and CIC mutant expressing vectors, and of stable cell lines.

The two cDNA clones expressing human untagged CIC and epitope tagged CIC were cloned into the pcDNA4/TO tetracycline regulated vector (Santa Cruz, T-Rex system). The strategy for generating the CIC dominant negative protein was based on studies performed in Dr. Kaplan's laboratory, showing that replacement of R181 and R189 of yeast CIC with cysteine residues abrogates citrate binding and/or export (see references in the main text and Walters et al., 2004). N185 within this region was also shown to stabilize citrate interaction. The equivalent residues of human CIC at position K190, N194 and R198 were replaced with C190, N194 and C198 by employing site directed mutagenesis, using the following primer forward: 5'GTCCTGTGTCAGGGCTCGAACCAGGCCATCTGCTT 3'. The cDNAs from wild-type and mutant CIC were then transfected in H1299 or 293T cells expressing the *tet* repressor and stable clones were obtained by double antibiotic selection with zeocyn and blasticidin, as per manufacturer instructions. Clones were isolated and first examined for CIC expression by employing both immuno-fluorescence and immuno-blot assays. Typically two-to-three clones showing homogenous levels of expression (i.e., more than 80% of cells expressing CIC proteins) were pooled together and used for further studies. After a few number of passages, CIC-DN expressing cells consistently showed a progressive reduction of CIC expression levels. Therefore, several experiments were performed with epitope Flag-CIC-DN expressing cells, where the presence of exogenous CIC could always be identified. The Flag does not interfere with mitochondrial localization or function.

## Quantification of metabolites, ATP, oxygen, ROS, MMP and mitochondrial mass.

The concentration of citrate, lactate, isocitrate, and malate were assessed using specific kits from BioVision. Cellular ATP levels were determined via the ATP kit (Promega), while oxygen consumption rates were measured using the BD oxygen biosensor systems (OBS) from BD Bioscience. Triplicate samples of 50,000 cells were seeded onto 96-well OBS plates. The number of cells was determined at each time point by sampling cells seeded into side-by-side plates. Fluorescence was measured from the bottom of the well every 24 h and measurements were normalized by subtracting the reading from the same well prior to the addition of the cells (blank). For measurement of ROS we used H2DCFDA (Invitrogen). Mitochondrial mass was assessed by using mitotracker green (Invitrogen), as per manufacturer instructions, or Nonyl Acridine Orange at a final

concentration of 300 nM, followed by analysis by flow cytometry. Mitochondrial Membrane Potential (MMP) was studied with the JC1 assay kit from Cayman.

### Mitochondria isolation and cellular sub-fractionation

Mitochondria and cytoplasmic fractions were prepared by using a protocol available from Abcam (http://www.abcam.com/index.html?pageconfig=resource&rid=11473), with some modifications. Cells were collected by centrifugation in PBS for 5', and washed once in 10 packed cell volumes of PBS. The resulting pellet was re-suspended in 5 packed cell volumes of mitochondria buffer A (250 mM Sucrose; 20 mM HEPES (7.4); 10 mM KCl ,1.5 mM MgCl2;1 mM EDTA;1 mM EGTA 0.5 M, supplemented with freshly added PMSF and 1 mM DDT). Cell lysates were passed through a 25 G needle 10 times, then 5 times through a 20 G needle, followed by incubation on ice for 10-15 minutes. Extracts were centrifuged at 720 G for 5 minutes. The supernatant was centrifuged again at 10000 G and the derived pellet (the mitochondrial fraction) was washed three times in buffer A, centrifuged again and re-suspended in RIPA buffer. The supernatant, (containing cytosolic and membrane fractions) was centrifuged multiple times to eliminate particulate material, and was used as the cytosolic fraction.

### Digitonin permeabilization, limited tryptic digestion of mitochondria and immuno-blots.

Mitochondria obtained from the last centrifugation step of the protocol described previously were resuspended in 50 volumes of mitochondria buffer A and equalization was performed on 1/10 of these fraction with a Bradford method. Equal aliquots of these mitochondria received different concentrations of digitonin and/or trypsin, and were subsequently incubated at 30 $^{o}$C for 30 minutes. Samples were centrifuged at 10000 for 15 minutes and the supernatant of these reactions was used for immuno-blot analysis. For analysis of CIC levels in immuno-blot from unfractionated samples or from mitochondria after fractionation, samples were suspended in RIPA buffer (0.1% SDS; 50 mM Tris·Cl, pH 6.8;150 mM NaCl; 0.5% Nonidet P-40; 2 mM EDTA) supplemented with a protease inhibitor mixture (Roche), as well as with TSA (500 µM) and with 10 mM N-ethylmalemide (NEM) and 5 mM DTT or Betamercaptoethanol. In experiments where we used tumor specimens from animals or humans, samples were first fragmented in liquid nitrogen and then extracted in RIPA buffer. SDS-PAGE electrophoresis was performed on 12-20% gradient gels (Novex), followed by transfer onto PVDF membranes (Milipore). We use 10% horse serum in PBS for as a blocking solution as well as for dilution or all antibodies. Incubation with primary antibodies was typically overnight at room temperature. Chemiluminescence was performed with the WestPico system (Pierce).

### Cellular proliferation assays and flow cytometry.

The proliferative capacity of cells was assessed by plating cells in a duplicate or triplicate at a concentration of 10,000 cells/well. Treatments with vehicle control, BTA or with tetracycline were applied at the time of plating and viable or dead cells were measured after 48-72 hours with trypan blue exclusion. For flow cytometry cells were collected and fixed in 70% ethanol for a minimum of 2 hours, after which propidium iodide was added. Cell cycle analysis was performed by the flow cytometry facility at LCCC, Georgetown University.

### Immuno-fluorescence.

Cells were plated on glass coverslips, treated with BTA or tetracycline, then fixed with 4% paraformaldehyde in PBS. Permeabilization was carried out with with 0.1% Triton X-100 in PBS for 10 min, followed by incubation with blocking solution (1% BSA in PBS). All antibodies were diluted in blocking solution. After incubation with primary antibodies cells were incubated with the appropriate secondary antibodies: donkey anti-mouse or anti-rabbit Alexa488 and Alexa566 fluoro-conjugated antibodies (Molecular Probes, Invitrogen). The coverslips were washed with PBS, nuclei were stained with DAPI and then mounted on slides with ProLong Gold anti-fade reagent (Invitrogen). Images were captured with an Axiovert 200M fluorescence microscope (Zeiss), equipped with an AxioCam MRm camera. The Z-stack images were obtained with 40x, 63x or 100x oil immersion objectives and, in some cases, were subjected to deconvolution with the Axio Vs40 software (Zeiss). Quantification of immune-fluorescence experiments was performed with the *Image J* software, as per manufacturer instructions.

## Mitochondrial enzyme assays.

Complex I activity was assayed in 10 mM Tris/HCl buffer (pH 7.4) containing 50 mM KCl, 1 mM EDTA, 125 $\mu$M NADH, 2 mM KCN and 300 nM antimycin A (Buffer A). After addition of disrupted mitochondria, ubiquinone-1 (50 $\mu$M) was added and the rate of disappearance of NADH was monitored at 340 nm. Rotenone (20 $\mu$M) was then added to set of samples. Activity was calculated as percentage of substrate used in the reaction after subtraction of the rotenone-insensitive fractions. The concentration of BTA used for these experiments was between 1 and 2 mM and treatment was overnight. Cells were scraped from the plate in the same reaction buffer (Buffer A, excluding NADH and inhibitors) and cell extracts were prepared by tree cycles of freeze and thaw in liquid nitrogen for three times. Equalization was performed with a Bradford-based assay. Typically 100 ug of total cell extracts were used in the reaction. Complex II activity was assayed in 50 mM $KH_2PO_4$ buffer (pH 7.4) containing 100 $\mu$M EDTA, 0.01% Triton X-100, 20 mM potassium succinate, 2 mM KCN, 10 $\mu$M rotenone and 300 nM antimycin A (Buffer B). After a 5 min preincubation at 30 °C to activate complex activity, ubiquinone-2 (50 $\mu$M) and 2,6-dichlorophenol-indophenol (DCIP; 75 $\mu$M) were added and the reduction of DCIP was measured at 550 nM. The background rate was measured after the addition of thenoyltrifluoroacetone (200 $\mu$M). Treatments, calculations and preparation of cell extracts was similar as described for Complex I, but cells were extracted in Buffer B.

## NMR Spectroscopy.

293T cells were plated at 20% confluency. The cultures were fed with glucose free media supplemented with 10 mM D-[1,6-$^{13}C_2$] glucose. BTA (1 mM) and tetracycline were added to the media and cells were harvested 12-16 hours thereafter. Cells were counted and equalized at $5X10^6$. Labeled and unlabeled control samples were ultrasonicated in the mixture of PBS and methanol (0.3 and 0.4 ml, respectively) for 1 min on ice and then mixed with chloroform (0.8 ml). After vigorous shaking, the samples were centrifuged at 2,000 x g for 5 min and the two layers (aqueous and organic layers) were separated. The lipid extraction step was repeated twice, and all the organic extracts were combined. Both organic and aqueous extracts were concentrated to dryness and reconstituted in chloroform-d and $D_2O$, respectively. The NMR measurements were performed with Bruker DRX Ultra-shield 700 MHz system equipped with a 1.7 mm micro-probe, and a temperature controller (set to 10°C). The NMR spectra were acquired and processed with Topspin 3.0 (Bruker Biospin)

## Mice and tumors.

To produce tumor xenografts $5 \times 10^6$ cells were resuspended in PBS and injected subcutaneously in the flanks of female nude mice. For drug treatments mice were randomized to receive either PBS or a PBS solution of BTA at a concentration 26 mg/kg which was administered via intra-peritoneal route three times a week. Mice were pre-treated twice prior to the inoculation of tumor cell lines. Once detectable tumors started to form, their size was measured with a caliper in three dimensions. Serial measurements were made at two-three day intervals after the identification of the initial cellular mass to determine growth curves *in vivo*. Tumor volume was calculated using the formula for a prolate spheroid: volume = (4/3) x $a^2b$, where $a$ is the width and $b$ is the length. All animals were sacrificed when the tumors exceeded 1.5 cm. At the completion of experiments, tumors were excised, weighed and statistical significance of differences in tumor volume were made using two factor repeated measures analysis of variance followed by Fisher's last significant difference test for multiple comparisons. The trial administration of BTA to explore its toxic effects, was conducted on 8 non immuno-compromized mice, randomized in two groups and injected as described before for five consecutive months. Animals were monitored once a week for the presence of signs of disease, particularly neurological disturbances or weight loss, and they were weighted periodically. All animal studies were approved by the Georgetown University Institutional Animal Care and Use Committee.

## CIC zebrafish methods.

### *Animals.*

Zebrafish (*Danio rerio*) were raised, maintained and crossed as described (Westerfield et al., 1995). All procedures were in accordance with NIH guidelines on the care and use of animals and were approved by the Georgetown University Institutional Animal Care and Use Committee.

***Morpholino oligonucleotide (MO) injections.***

A translation blocking anti-*slc25A1* (CIC) morpholino oligonucleotide (MO) was designed and synthesized by Gene-Tools, LCC, with the following sequence: CIC MO, 5'-CATGTTTGCTGATGTTTTGAGCGTA. The translational start site is underlined. The Standard Control MO from Gene Tools was used as control. Solutions consisting of 4ng/nl MO in dH20, plus 0.5% tetramethyl rhodamine dextran, or .05% phenol red, were microinjected into one to four cell stage embryos.

***Zebrafish embryo extracts.***

Extracts were prepared from zebrafish embryos at 24 hours post fertilization (hpf) following the protocol for dechorionating and deyolking embryos (Westerfield M. 1995). Briefly, chorions were removed, in batches of 50 embryos, by limited digestion in 1mg/ml pronase, followed by three rinses in ice cold Ringer's solution. The embryos were then transferred to cold Ringer's containing 1mM EDTA and 0.3 mM phenylmethylsulfonylfluoride (PMSF) and deyolked by titurating through a 200ul micropipette tip. The deyolked embryos were rinsed in Ringer's solution and either stored at -70C or used for further analysis. For immuno-blot experiments, embryos were typically lysed in RIPA buffer, and a concentration of approximately 40 g was used to detect CIC, mitofilin or LC3 with specific antibodies (these latter two, were raised against human recombinant protein). The extent of homology between human and zebrafish CIC, as well as the epitope recognized by the anti-CIC antibody is shown in Figure S7. A similar analysis was performed in the case of zebrafish LC3 and mitofilin, which also exhibit a high degree of similarity with the human proteins. For extraction of DNA, a commercially available kit (Quiagen) was used.

## Statistical analysis.

Data are expressed as means ± standard deviations (SD). The two-tailed Student *t* test was used for all statistical analysis of experiments presented and Excel was used for statistical calculations. Significant differences are indicated using the standard Michelin Guide scale ($P < 0.05$, significant; $P < 0.01$, highly significant; $P < 0.001$, extremely significant).
